# Band Alignments, Electronic Structure, and Core-Level Spectra of Bulk Molybdenum Dichalcogenides (MoS_2_, MoSe_2_, and MoTe_2_)

**DOI:** 10.1021/acs.jpcc.2c05100

**Published:** 2022-12-01

**Authors:** Leanne A. H. Jones, Zongda Xing, Jack E. N. Swallow, Huw Shiel, Thomas J. Featherstone, Matthew J. Smiles, Nicole Fleck, Pardeep K. Thakur, Tien-Lin Lee, Laurence J. Hardwick, David O. Scanlon, Anna Regoutz, Tim D. Veal, Vinod R. Dhanak

**Affiliations:** † Stephenson Institute for Renewable Energy and Department of Physics, University of Liverpool, LiverpoolL69 7ZF, U.K.; ‡ Department of Chemistry, University College London, 20 Gordon Street, LondonWC1H 0AJ, U.K.; § Diamond Light Source Ltd., Diamond House, Harwell Science and Innovation Campus, Didcot, OxfordshireOX11 0DE, U.K.; ∥ Stephenson Institute for Renewable Energy and Department of Chemistry, University of Liverpool, LiverpoolL69 7ZF, U.K.

## Abstract

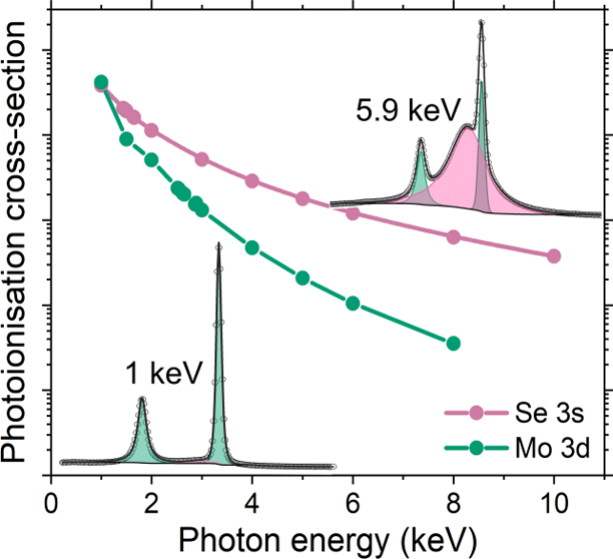

A comprehensive study of bulk molybdenum dichalcogenides is presented with the use of soft and hard X-ray photoelectron (SXPS and HAXPES) spectroscopy combined with hybrid density functional theory (DFT). The main core levels of MoS_2_, MoSe_2_, and MoTe_2_ are explored. Laboratory-based X-ray photoelectron spectroscopy (XPS) is used to determine the ionization potential (IP) values of the MoX_2_ series as 5.86, 5.40, and 5.00 eV for MoSe_2_, MoSe_2_, and MoTe_2_, respectively, enabling the band alignment of the series to be established. Finally, the valence band measurements are compared with the calculated density of states which shows the role of p-d hybridization in these materials. Down the group, an increase in the p-d hybridization from the sulfide to the telluride is observed, explained by the configuration energy of the chalcogen p orbitals becoming closer to that of the valence Mo 4d orbitals. This pushes the valence band maximum closer to the vacuum level, explaining the decreasing IP down the series. High-resolution SXPS and HAXPES core-level spectra address the shortcomings of the XPS analysis in the literature. Furthermore, the experimentally determined band alignment can be used to inform future device work.

## Introduction

Although studied in the 1960s, the full potential of the transition-metal dichalcogenides (TMDs) was not initially realized. However, the development of graphene reinvigorated interest in them, particularly in mono- and few-layer forms. Most TMDs possess the honeycomb structure that graphene is well known for; however, their electronic properties differ greatly. The TMDs experience a vast range of electronic properties including semiconducting, semimetallic, and metallic behavior, which opens a large range of uses for these materials.

**Figure 1:**
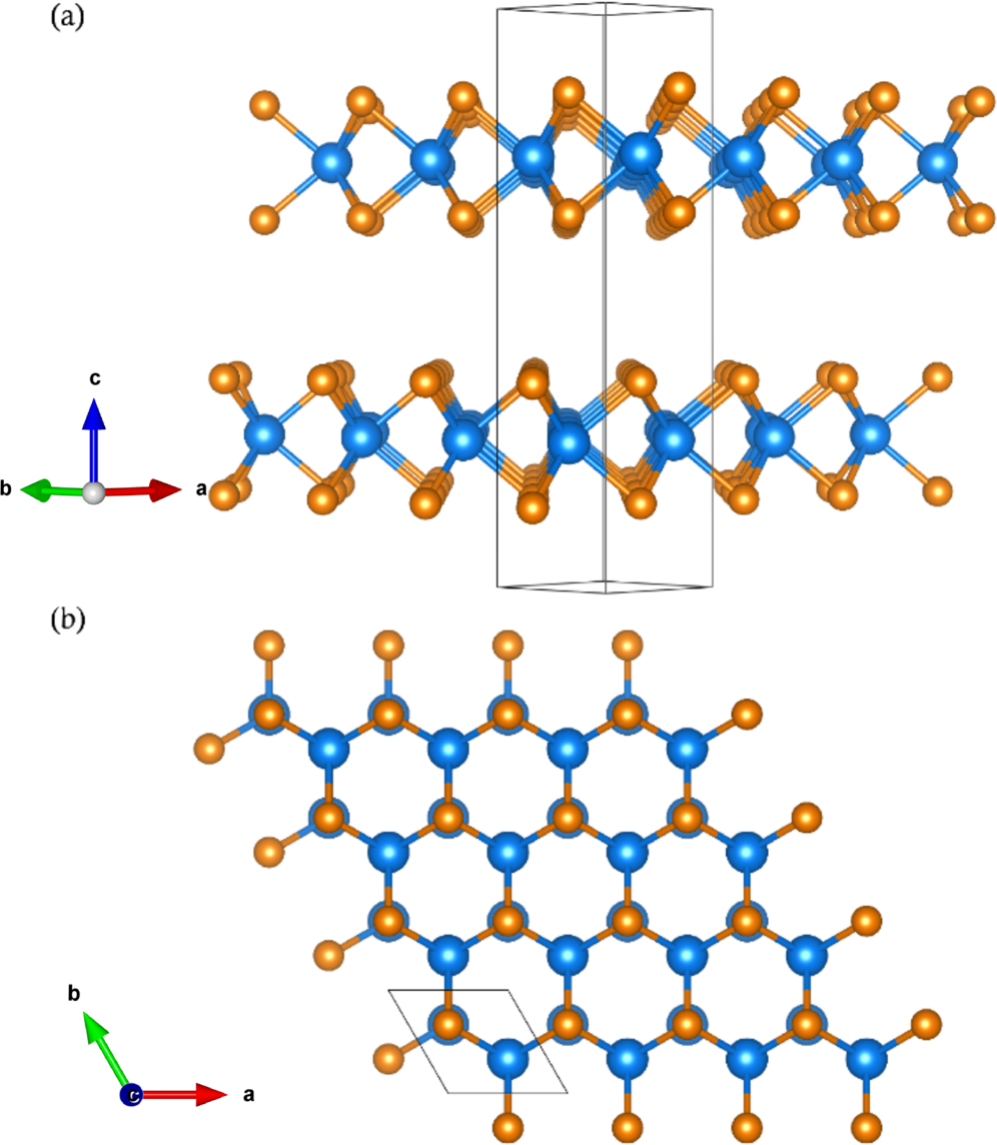
2H structures of MoS_2_ representative of all MoX_2_ showing the configuration of the molybdenum (blue) and chalcogenide (orange) atoms. (a) Layered nature and the trigonal prismatic coordination of the chalcogen atoms around the Mo atom. (b) Hexagonal structure within the layers. The images were prepared using the VESTA software package.^7^

A series that belongs to the TMD family is the molybdenum dichalcogenides with the chemical composition MoX_2_ (X = S, Se, and Te). The most stable phase of these dichalcogenides is the semiconducting 2H phase with the *P*6_3_/*mmc* space group, which is presented in Figure 1.^1−3^ In the 2H phase, the metal atoms are in a trigonal prismatic coordination where they are covalently bonded to six chalcogen atoms in the form of two tetrahedrons in the +*c* and −*c* directions; this completes a monolayer of MX_2_. In bulk form, monolayers are held together by van der Waals interactions. It should be noted that the MoX_2_ series can be found in other stable phases; for example, MoS_2_ and MoSe_2_ can also be found in the octahedral 1T phase^4,5^ and MoTe_2_ in the orthorhombic T_d_ phase as well as the distorted octahedral 1T’ phase.^6^ The effect of the trigonal prismatic coordination of the chalcogen atoms on the electronic structure is the splitting of the transition-metal d orbitals as predicted by crystal field theory. The resulting orbitals consist of three degenerate levels: a singlet, denoted as “*a*”, which consists of the d_*z*^2^_ orbital, and two doubly degenerate levels denoted as *e*^′^ and *e*^″^, which consist of the d_*xy*_ and d_*x*^2^–*y*^2^_ and the d_*yz*_ and d_*xz*_ orbitals, respectively. The *a* level has the lowest energy, followed by *e*^′^ and *e*^″^. This is because the *a* orbital consists of the d_*z*^2^_ orbital which projects out of the plane and into the van der Waals gap, meaning that it does not interact strongly with the chalcogen orbitals.

The van der Waals interactions between the layers and the consequently large specific surface area make TMDs attractive for energy storage, for example, in electrochemical capacitors,^8^ and as the negative electrode material for lithium-ion batteries.^9−11^ The high surface-to-volume ratio also makes them useful for sensors, including transistor-based sensors for gas, chemical, and bio-sensing.^12−16^ The ability to exfoliate these materials makes them attractive for ultrasmall and flexible transistors.^17,18^ The most interesting aspect of the TMDs is their layer-dependent properties, making them suitable for many optoelectronic devices such as solar cells, photodetectors, and light-emitting diodes.^19−25^ MoS_2_, in particular, has sparked large interest for these applications. Recently, MoTe_2_ has been identified as a candidate for field-effect transistors,^26^ but is in the early stages of understanding the physical mechanisms that limit device performance. Although many device studies focus on monolayer TMDs, achieving large-scale high-quality monolayers is very difficult and often limits the application. Methods of altering the band gap from indirect to direct for bulk TMDs have recently been investigated.^27^ Furthermore, bulk MoS_2_ has been found as a promising candidate for photovoltaic devices using an environmentally friendly method of depositing MoS_2_ thin films.^28^ MoSe_2_ has had a lot of interest for use in energy storage devices as it has larger interlayer spacing compared to that of MoS_2_. Because of the larger spacing, it has the potential to accommodate larger ions such as Na.^2^ Moreover, MoSe_2_ has also been studied for catalysis, including the hydrogen evolution reaction as well as for CO_2_ reduction.^2^ MoTe_2_ has a comparable interlayer spacing to MoSe_2_ and thus has also been considered as an anode material candidate for Li-ion storage.^10,29^ Additionally, bulk MoTe_2_ has also been studied for two-dimensional (2D) gas sensors^30,31^ as well as for photodetectors.^32,33^ Hence, a good understanding of the bulk electronic structure is beneficial for the development of applications of bulk TMDs.

A number of theoretical investigations of the electronic structure of these materials exist using different methods.^34−41^ Both theory-only studies and angle-resolved photoemission spectroscopy (ARPES) studies exist. However, no studies report a comprehensive comparison of the theoretical density of states (DOS) with valence band X-ray photoelectron spectroscopy (XPS) measurements. Only one study compares XPS/ultraviolet photoelectron spectroscopy (UPS) measurements of the MoS_2_ and MoSe_2_ valence bands to calculations, which includes limited discussions of the band gaps and ionization potentials (IPs) obtained from theory.^34^ No discussion of the partial density of states, which is essential to understand the nature of the bonding within these materials, was included. Beyond valence states, the core-level analysis in the literature is often filled with erroneous modeling, in particular for MoSe_2_, where the Se 3s peak that lies in between the two Mo 3d doublet peaks is often overlooked. This is discussed further in the Core-Level Spectra section.

Therefore, this paper presents high-resolution soft and hard X-ray photoelectron spectroscopy (SXPS and HAXPES, respectively) data of bulk Mo dichalcogenides to investigate their electronic structure. The valence band spectra measured by soft and hard XPS are compared to density functional theory calculations of the occupied density of states. Analysis of the main core-level spectra of molybdenum (Mo 3d) and main chalcogen core levels (S 2p, Se 3d, and Te 3d) are investigated.

## Experimental and Computational Details

The crystals used for this study were grown by HQ graphene using chemical vapor transport and have greater than 99.995% purity.

For the Raman measurements, a Renishaw inVia Raman microscope was used in backscattering geometry. The laser wavelength was 532 nm, with a power of 0.2 mW, and was focused through an inverted microscope (Leica) via a 50× objective lens. The laser spot diameter was 1–2 μm.

### Laboratory-Based XPS

Laboratory-XPS measurements were utilized to measure the ionization potential of the dichalcogenide series. These measurements were conducted using a monochromatic Al Kα (*h*ν = 1486.6 eV) SPECS X-ray source operated at 250 W. Emitted photoelectrons were measured using a PSP Vacuum Technology hemispherical electron energy analyzer with a mean radius of 120 mm using a pass energy of 10 eV. All measurements were performed in an ultrahigh vacuum chamber with a base pressure of 2 × 10^–10^ mbar. All binding energies were measured with respect to the Fermi edge of a clean polycrystalline silver reference sample. The resolution of the spectrometer was found to be 0.40 eV by fitting the Fermi edge with a Fermi–Dirac function convolved with a Gaussian function. Binding energies are determined with an uncertainty of ±0.05 eV. The measurements were conducted following *in situ* exfoliation at a pressure of 1 × 10^–9^ mbar to prepare surfaces with minimal contamination. The *in situ* exfoliation was achieved by mechanical exfoliation using carbon tape. The carbon tape was pressed onto the flattened surface of the crystal and pulled away, exfoliating the top few layers.

Hard and soft X-ray photoelectron spectroscopy measurements were conducted at the I09 beamline^42^ at Diamond light source (DLS), Oxfordshire, U.K. These measurements were conducted to measure soft (1.0 keV) and hard (5.9 keV) X-ray photoelectron spectra on the same spot on *in situ* cleaved surfaces of the MoX_2_ series. The cleaving was done at a base pressure of 1 × 10^–11^ mbar. The crystals were mounted onto a copper plate using a silver-based epoxy which was cured at 120 °C for ∼30 min. A peg was then mounted onto the sample using the same epoxy, and once *in situ*, the peg was tapped with a wobble stick, removing the top few layers exposing a clean surface. The hard X-rays used were defocussed with an energy of 5.9 keV selected by a double-crystal Si(111) and Si(004) channel-cut monochromator. The soft X-rays were also defocussed with a photon energy of 1.0 keV. The soft X-ray energy was selected using a plane grating monochromator. The energy of 1.0 keV was chosen to ensure no overlap of Auger lines with the main core-level peaks. The experimental resolution was determined by measuring and fitting the Fermi edge of a polycrystalline gold sample with a Gaussian broadened Fermi–Dirac distribution and was determined to be 0.28 and 0.26 eV for the HAXPES and SXPS measurements, respectively. Binding energies are determined with an uncertainty of ±0.03 eV. The system uses a VG Scienta EW4000 electron analyzer with a ±28° acceptance angle.

### Hybrid Density Functional Theory

All calculations in this work were performed using the Vienna *ab initio* Simulation Package (VASP)^43−46^ under the framework of density functional theory (DFT), with the core valence electrons interaction described by the projector augmented wave method.^47^ The initial bulk structures of the molybdenum dichalcogenides (MoX_2_, X = S, Se, Te) were obtained from experimental data in the inorganic crystal structure database (ICSD).^48−52^ We include the 4d, 4p, and 5s valence orbitals for Mo; 3s and 3p orbitals for S; 4p, 4d, and 3d for Se; and finally the 5s, 5p, and 4d for the Te valence orbitals. The Perdew–Burke–Ernzerhof (PBE) generalized gradient approximation functional^53^ was used in all geometry optimizations with the correction for van der Waals dispersion (DFT-D3),^54^ and a combination of 400 eV plane-wave energy cutoff and Γ-centered *k*-point mesh of 8 × 8 × 8 was sufficient for all MoX_2_ to converge within 10^–5^ eV per atom. For the geometry relaxation, the energy cutoff was increased by 30% to account for Pulay stress introduced by the finite basis sets, and the structures were considered fully relaxed when the force on all atoms is less than 0.01 eV Å^–1^. Electronic properties such as density of states and band structures of the relaxed structures were calculated using the HSE06 functional with the spin-orbital coupling effect included.^55,56^ A screening parameter of 0.11 Bohr^–1^ is used to determine the short-range cutoff of 25% Hartree–Fock exchange. The band structures were plotted using the sumo package.^57^ A more in-depth discussion of the functional used can be found in the Supporting Information (SI) along with Figure S9.

## Phase Purity

**Figure 2:**
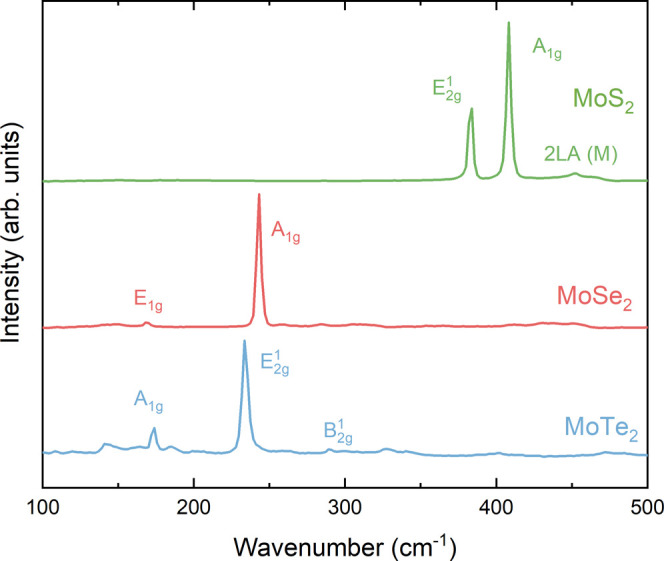
Raman spectra for the three molybdenum dichalcogenides with the main modes labeled.

Raman spectroscopy was used to verify the phase purity of the crystals used for SXPS and HAXPES. Figure 2 shows the Raman spectra for the three molybdenum dichalcogenides. The main features are labeled on the graph. Because all three chalcogenides have the *D*_6*h*_^4^ space group, they have four Raman-active modes, E_1g_, A_1g_, E_2g_^1^, and E_2g_^2^,^58,59^ where the modes present depend on the laser wavelength^58^ and crystal orientation. Starting with MoS_2_ (green spectrum in Figure 2), the main Raman modes seen are at 383 and 406 cm^–1^ which are the E_2g_^1^ and A_1g_ modes, respectively, with a small intensity as 452 cm^–1^ which agrees with previous reports.^60,61^ For MoSe_2_, one strong mode is seen at 243 cm^–1^ which is the A_1g_ mode and a less intense mode at 169 cm^–1^ corresponding to the E_1g_ mode again, agreeing with previous literature.^58,59^ Finally, MoTe_2_ has two strong features, one at 234 cm^–1^, which is that of the E_2g_^1^ mode, and the second at 174 cm^–1^, which corresponds to the A_1g_ peak; these values also agree with what has been reported previously.^62,63^ The weaker features are also seen in bulk 2H-MoTe_2_ but are often not reported; these correspond to the E_1g_ and B_2g_^1^ modes.^64^ No peaks corresponding to Mo oxides were detected.^65^ These Raman spectra show the high quality and phase purity of these crystals.

## Hybrid Density Functional Theory

In Table 1, the lattice parameters acquired from the calculations are compared to literature experimental values and show good agreement within 5% to the experimental values.^50,52,66−68^ It is important to note that one source of difference between the theoretical and experimental values is due to the 0 K temperature of the hybrid DFT calculations compared to the experiments being performed at room temperature.

**Table 1:** Calculated Lattice Parameters of This Work Compared to Experimental Literature Values

MoS_2_	this work	ref (66)	ref (50)
*a* (Å)	3.162		3.15
*c* (Å)	12.313		12.30
*c* (a)	3.894	3.892	3.905

MoSe_2_	this work	ref (66)	ref (67)
*a* (Å)	3.294		3.288
*c* (Å)	13.002		12.391
*c* (a)	3.894	3.947	3.769

MoTe_2_	this work	ref (52)	ref (68)
*a* (Å)	3.518	3.519	3.5139
*c* (Å)	14.069	13.964	13.964
*c* (a)	3.999	3.968	3.974

**Figure 3:**
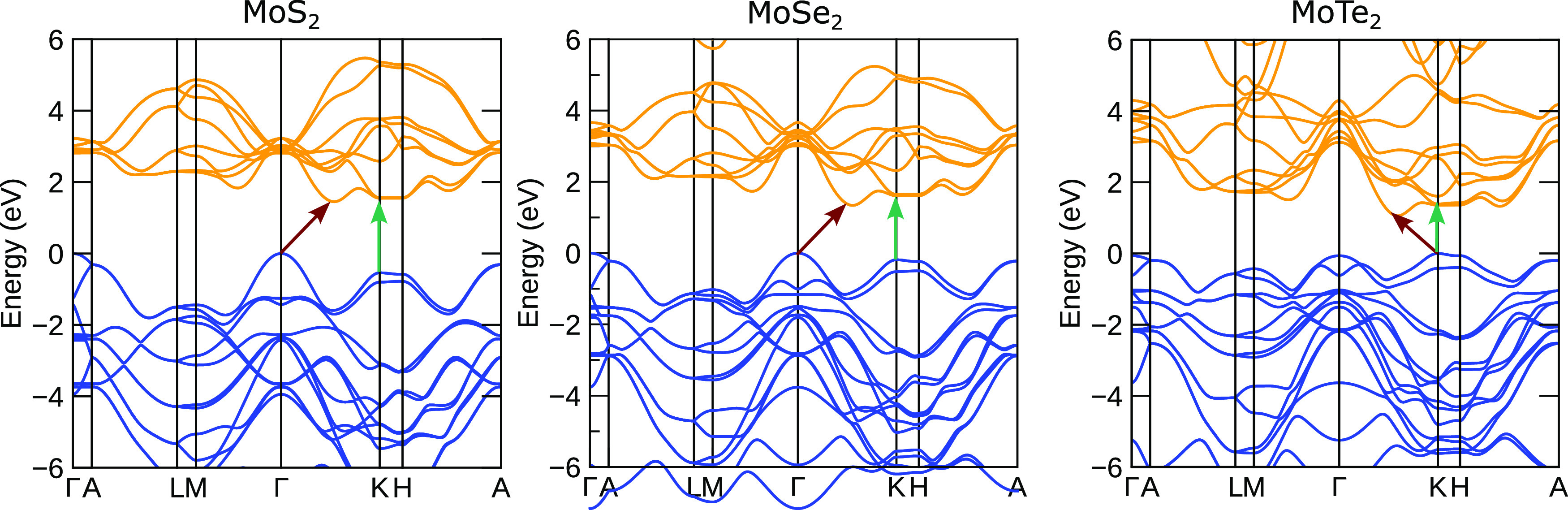
Calculated band structures of bulk MoS_2_, MoSe_2_, and MoTe_2_. The lowest indirect (direct) band gaps are indicated by red (green) arrows.

The theoretical band structures of the three chalcogenides are shown in Figure 3. As is well known for MoS_2_ and MoSe_2_, the smallest band gap is indirect with the valence band maximum (VBM) at the Γ point and the conduction band minimum (CBM) in between the Γ and the *K* point. For MoTe_2_, the CBM is located in the same place, however, the VBM is located at the *K* point. The calculated indirect band gap values were 1.450, 1.338, and 1.057 eV for MoS_2_, MoSe_2_, and MoTe_2_, respectively. Experimentally, the room temperature indirect band gaps of the bulk molybdenum dichalcogenides have been investigated using photoacoustic spectroscopy and were determined to be 1.37 eV for MoS_2_, 1.25 eV for MoSe_2_, and 0.89 eV for MoTe_2_.^69^ The lowest direct band gap values obtained from the present calculations were 2.088, 1.794, and 1.354 eV for MoS_2_, MoSe_2_, and MoTe_2_, respectively. These are overestimated in comparison to the experimental values of 1.87, 1.56, and 1.06 eV for MoS_2_, MoSe_2_, and MoTe_2_, respectively, determined by modulated photoreflectance spectroscopy.^69^ The direct band gaps of the dichalcogenides have also been investigated at low temperatures. These values should in theory be more comparable with the 0 K theoretical values presented here. Bulk MoS_2_ exhibits a direct band gap at 1.932 eV measured at 10 K using photoreflectance spectroscopy.^70^ 16 monolayers of MoSe_2_ were estimated to have a 0 K band gap of 1.50 eV from the extrapolation of temperature-dependent ellipsometry data.^71^ For MoTe_2_, the direct band gap at 4.5 K was determined to be ∼1.15 eV.^72^ For the indirect band gap however, low temperature values are a lot less common within the literature. The indirect band gap of MoS_2_ and MoSe_2_ was found by optical transmission to be 1.17 and 1.11 eV at 70 K, respectively.^73^ A report of the indirect band gap of MoSe_2_ at 12 K gives a value of 1.165 eV.^74^ All band gaps discussed here can be found in tabulated form in Table S1 in the Supporting information (SI). A reason for the small overestimation of the direct and indirect band gaps by DFT could be due to the treatment of the van der Waals forces coupled with the HSE06 functional used in these calculations. There are several studies where accurate band gaps have been obtained using different calculation methods such as GW^34^ and the GvJ-2e method.^40^ However, this paper focuses on the electronic structure rather than the band gap.

## Core-Level Spectra

**Figure 4:**
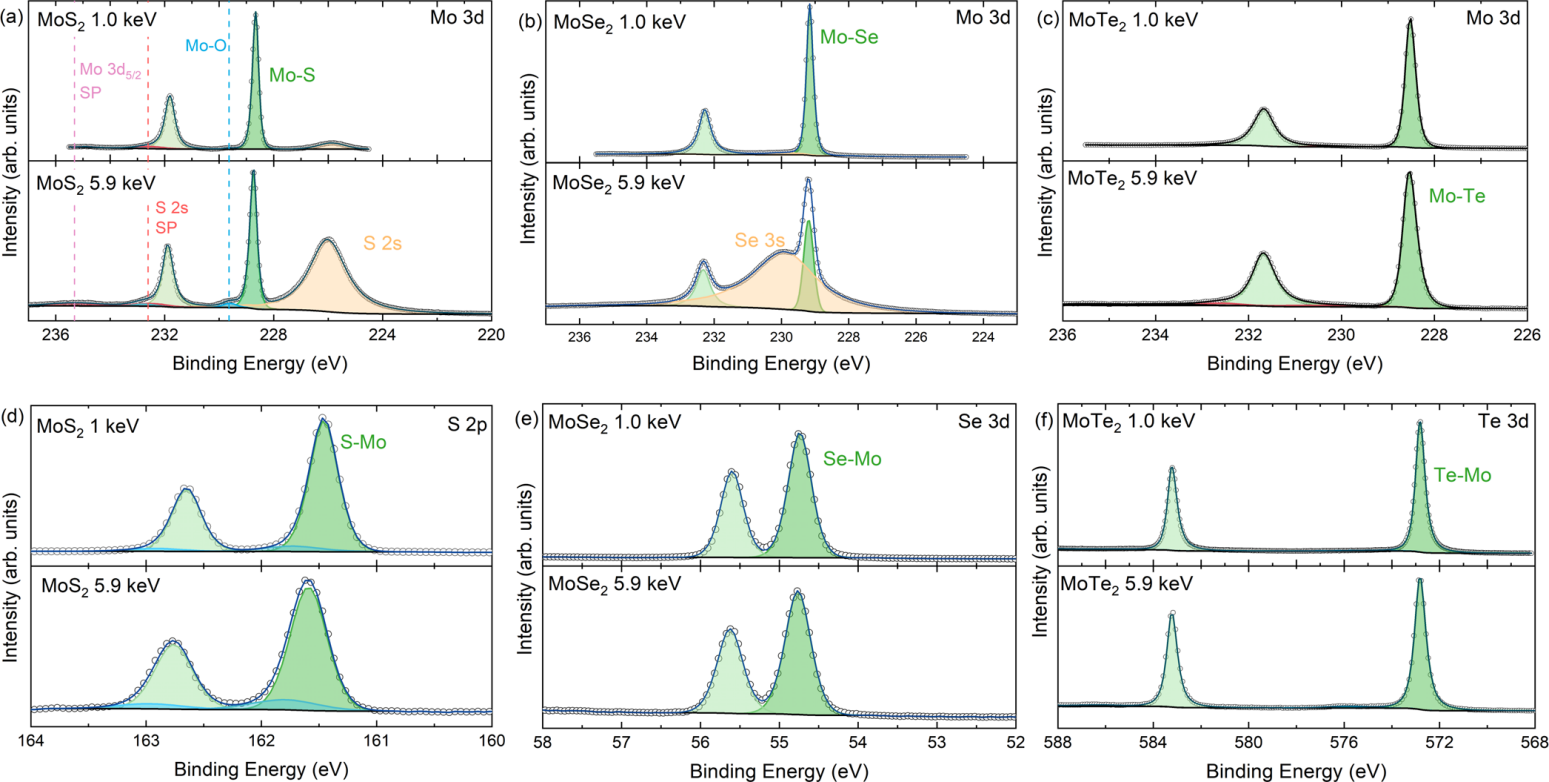
Core-level spectra of the Mo dichalcogenide series where (a) and (d) are the Mo 3d and S 2p core levels of MoS_2_, (b) and (e) are the Mo 3d and Se 3d core levels of MoSe_2_; and (c) and (f) are the Mo 3d and Te 3d core levels of MoTe_2_. All core levels were taken at two photon energies, 1.0 and 5.9 keV. *SP denotes surface plasmon.

The Mo 3d core levels are shown in Figure 4a–c, where the spin–orbit splitting of the Mo 3d core-level fit was constrained at 3.15 eV for all three dichalcogenides. Furthermore, the area ratios of the fit were also constrained, according to the ratio of the interpolated photoionization cross sections of the orbitals calculated by Scofield.^75^ This becomes very important at higher photon energies due to the  component having a smaller radial extension in comparison to that of the  component. In turn, when the photon energy is increased the overlap between the continuum of the  orbital is larger than that of the  orbital, which means the cross section is greater and thus the area ratio will be different from the conventionally used statistical ratio of 3:2. This has been shown to be prominent in some d orbitals^76^ and is therefore taken into account here. The values used for the area ratios of the fit can be found in Table S2 in the Supporting Information (SI). However, the full width at half-maxima are not constrained as the Coster–Kronig effect causes additional broadening of the 3d_3/2_ peak with respect to the 3d_5/2_.^77−80^

There are many reports of core-level measurements of the MoX_2_ series. However, there are many discrepancies when it comes to peak fitting and analysis. For MoS_2_, there are no overlaps between the core levels which makes fitting easier in comparison to MoSe_2_ where the Mo 3d and Se 3s regions overlap. Despite this, some previous reports contain erroneous or incomplete fitting. These take the form of: (1) incorrect spin–orbit splitting constraints being used; (2) no area constraints applied; (3) incorrect lineshapes and lack of backgrounds shown; (4) the envelope of the core-level fit is not overlaid onto the data so that a clear comparison cannot be made or no fit is shown at all; (5) no binding energies are quoted.^81−86^Figure 4a presents the fitted Mo 3d and S 2s core levels of MoS_2_. Here, the cross section effect between the SXPS and HAXPES is highlighted, whereby between the 1.0 and 5.9 keV spectra, the S 2s cross section decreases relatively less than that of Mo 3d. The main Mo 3d_5/2_ core-level line is attributed to Mo bonded to S, and it is found at a binding energy of 228.66 and 228.74 eV for SXPS and HAXPES, respectively. These values are consistent with reported values when binding energy calibration is taken into account.^85,87^ For the fitting of the Mo 3d peaks, it can be seen that a second set of doublets was required with low intensity to achieve a good fit. This second peak at 229.61 and 229.63 eV for SXPS and HAXPES, respectively, is attributed to some surface oxide remaining after cleaving due to a rough surface. The binding energy of Mo(IV) oxide varies within the literature between 229.3 and 230.1 eV,^88^ which is in line with our binding energy values. A measurement of as-entered MoS_2_ can be seen in Figure S1 in the Supporting Information where a very strong Mo 3d oxide peak can be seen at 0.7 eV higher than the Mo–S peak. This is in line with the energy separation between the two components seen here for the *in situ* cleaved sample. Further discussion of the O 1s core level is presented in Figure S2 and related text. Two extra peaks are required to fit the data at ∼6.5 eV higher than the S 2s and Mo 3d_5/2_ in the HAXPES core levels which are attributed to energy loss from some of the Mo 3d photoelectrons to surface plasmon excitations that have been reported before for XPS of MoS_2_^89^ (zoomed-in version can be seen in Figure S3). The S 2p core level was also fitted and can be seen in Figure 4d. The doublet separation used here was 1.18 eV with an area ratio that can be found in Table S2 in the SI between the S 2p_3/2_ and 2p_1/2_. The binding energy of the S 2p_3/2_ was found to be 161.45 and 161.58 eV for SXPS and HAXPES, respectively. A weaker component at ∼0.3 eV above the main lines is also required to achieve a good fit. This component may be observable here due to the energy resolution being greater than for many reports. This weak component is not thought to be due to residual surface contamination as its relative intensity is stronger in the HAXPES measurement and the chemical shift is smaller than typically reported for contaminant peaks. A component at ∼0.3 eV higher binding energy than the main S 2p line has been observed previously for monolayer MoS_2_, where it was associated with some of the subsurface sulfur, close to the Au substrate.^90,91^ Clearly, the same interpretation cannot be made here as our data is from in situ cleaved bulk MoS_2_. We therefore tentatively associate the component with some kind of structural defect, possibly generated by cleaving.

There are differences of 0.08 and 0.13 eV for the Mo 3d and S 2p core levels, respectively, when measuring with 1 and 6 keV photons. For Mo 3d and S 2p, this can be attributed to some surface band bending resulting from the small contamination left at the surface. However, S 2p exhibits a slightly higher binding energy difference, which cannot be explained by surface band bending alone as both core levels have similar binding energies and so comparable photoelectron kinetic energies. To explain this difference in the photon energy dependence, recoil-induced binding energy shift is investigated.^92^ This phenomenon is more significant for light elements and when using higher photon energies as the photoelectron escapes with very large kinetic energy. As a result, the atom from which the photoelectron is emitted recoils to conserve momentum. The value for the recoil energy (Δ*E*) can be estimated using the equation: Δ*E* = *E*_K_(*m*/*M*) where *E*_K_ is the photoelectron kinetic energy, *m* is the mass of the photoelectron and *M* is the mass of the atom from which the photoelectron originates.^93^ For 6 keV photons, the S 2p recoil shift is estimated to be 0.098 and 0.031 eV for Mo 3d. The difference between these two recoil shifts is 0.067 eV which is close to the 0.05 eV difference between the 0.08 and 0.13 eV binding energy differences of the Mo 3d and S 2p SXPS and HAXPES peaks. This, therefore, shows that the greater difference of S 2p core-level binding energy with respect to Mo 3d can be explained by energy-dependent recoil-induced binding energy shift. The S 2s core-level binding energy position for SXPS and HAXPES is also consistent with this interpretation. The recoil shift effect is insignificant for MoSe_2_ and MoTe_2_, which do not contain low-mass S atoms.

The Mo 3d of MoSe_2_ region has been misreported the most, due to the overlapping Se 3s core level. The HAXPES measurement can be utilized to determine the energy separation between the Mo 3d_5/2_ and Se 3s, due to the photoionization cross sections. This can be seen in Figure 4b, where at 5.9 keV, the Se 3s dominates the spectrum and the peak position can be clearly determined. From this, the separation between the Mo 3d and Se 3s was found to be 0.7 eV. This was applied to the 1.0 keV spectrum as the Se 3s peak is not as well defined. The Se 3s intensity has been overlooked in many previous reports where the region was fitted by either adjusting the background to omit the intensity, not including the background at all, using inappropriate lineshapes or simply not acknowledging the missing intensity in the fit.^94−97^ Despite these, one study does mention the Se 3s core level,^98^ although when inspecting the fit of the other core levels, it is evident that no peak constraints have been used, which raises the question of whether peak constraints were used in any of the fitting. Here, the binding energy of the Mo 3d_5/2_ was found to be 229.15 and 229.19 eV at 1.0 and 5.9 keV, respectively. The Se 3d spectrum can be seen in Figure 4e, where a spin–orbit splitting value of 0.86 eV and the area ratios can be found in Table S2 in the SI. The Se 3d_5/2_ has a binding energy of 54.74 and 54.76 eV for SXPS and HAXPES, respectively.

Finally, MoTe_2_ has the fewest issues within the existing XPS literature. Here, the binding energy of the Mo 3d_5/2_ was found to be 228.52 and 228.54 eV at 1.0 and 5.9 keV, respectively. When inspecting the HAXPES measurement, extra intensity is apparent around the Mo 3d_3/2_ core level in comparison to the SXPS measurement. This is not thought to be due to contamination as the same spot was measured with 1.0 keV photons and there were no extra components needed for the fitting. Furthermore, the 1.0 keV photon energy measurement is more surface sensitive due to the escape depth of the electrons being lower and thus, if there were any features due to contamination they would be stronger in these measurements. Therefore, it is believed that the extra intensity is due to surface and bulk valence band plasmons of MoTe_2_. It has been reported that MoTe_2_ exhibits plasmon excitations with an energy of ∼20 eV.^99,100^ This is seen in the HAXPES survey where every peak has a strong plasmon peak at ∼20 eV high binding energy than the main core level, corresponding to kinetic energy loss to the plasmon excitations. It has also been reported that there are weak and broad electron energy loss features for energies between 3 and 8 eV^100^ in MoTe_2_. This would correspond to weak features at 3–8 eV above the main line which are seen in the 5.9 keV Mo 3d spectrum. For Te 3d_5/2_, the main core-level binding energy is 572.82 eV for both SXPS and HAXPES, which can be seen in Figure 4f. The Te 3d spectrum required a very small extra component for both the 1.0 and 5.9 keV data at higher binding energy. The high binding energy component at 575.87 eV, which is 3.1 eV above the main line, in the HAXPES measurement is attributed to oxide. This is corroborated by the as-entered XPS measurement which also exhibits a peak at ∼3.1 eV above the main line due to the presence of oxide. Furthermore, TeO_2_ has been reported to have a binding energy of ∼576 eV.^101,102^Table 2 shows the binding energies for the SXPS and HAXPES measurements of the main Mo and chalcogenide peaks.

**Table 2:** Binding Energies for the Main Core Lines Seen in Figure 4 at 1.0 keV (SXPS) and 5.9 keV (HAXPES)^a^

	Mo 3d_5/2_ (eV)	S 2p_3/2_ (eV)	Se 3d_5/2_ (eV)	Te 3d_5/2_ (eV)	
MoS_2_	228.66	161.45			SXPS
	228.74	161.58			HAXPES
MoSe_2_	229.15		54.74		SXPS
	229.19		54.76		HAXPES
MoTe_2_	228.52			572.80	SXPS
	228.54			572.80	HAXPES

a The uncertainties of the values are estimated to be ±0.03 eV.

## Work Function (WF), Ionization Potential, and Band Alignments

As well as the core-level measurements, valence band and secondary electron cutoff measurements were taken. These allow for the valence band maximum (VBM), work function (WF) and ionization potential (IP) values to be extracted. The VBM value can be determined using two methods, the first being the linear extrapolation method. This involves fitting the VB edge with a linear function and doing the same for the background and where these two lines intercept is the VBM value. The second is by shifting the theoretical density of states (DOS) to the leading edge of the valence band data and seeing how much it has been shifted, as the calculation has the VBM set at 0 eV. The first method is the most popular but there are some caveats to this technique to bear in mind: (1) if the material has a sharp onset of the density of states then the instrumental broadening dominates the VB edge which in turn skews the extrapolated value;^103^ (2) the constituents of the VB edge as the relative photoionization cross section can change the constituent of the valence band edge. An example of this is in MoSe_2_ and MoTe_2_ as the VB edge is dominated by the Mo d orbitals when measured by SXPS and by Se/Te p orbitals when measured by HAXPES, this is presented in Figure 6. Here, both methods were employed for SXPS and HAXPES to investigate the VBM value. This allows for the comparison of the values obtained between the two methods and furthermore, between SXPS and HAXPES. Table 3 displays the valence band maximum values for all three chalcogenides using the extrapolation and the “DOS fit” method. The fitting of the extrapolation method can be found in Figure S4, and the DOS fit method is described in Comparison of Theoretical Density of States and Experimental Valence Band Spectra section. For MoS_2_, MoSe_2_, and MoTe_2_, the values of both methods agree very well with each other.

**Table 3:** Valence Band Maximum Values for the MoX_2_ Series Using Both the Extrapolation Method and by Aligning the Hybrid DFT DOS to the Experimental Data for SXPS and HAXPES^a^

	extrapolation (eV)	DOS fit (eV)	
MoS_2_	0.1	0.1	SXPS
	0.1	0.2	HAXPES
MoSe_2_	1.2	1.0	SXPS
	1.1	1.0	HAXPES
MoTe_2_	0.7	0.8	SXPS
	0.8	0.8	HAXPES

a The uncertainties of the values are estimated to be ± 0.1 eV.

For MoS_2_, the determined valence band maximum position is very sensitive to where the extrapolation is fitted. As well as the main edge, there is a weak intensity just below this, which can be explained by comparing the experimental valence band to the band structure, shown in Figure S5. A low density of states at the VBM is seen in the band structure corresponding to the weak intensity at the VB edge. The top of the valence band consists of highly dispersive bands that have a weak contribution to the DOS compared with the flatter features at about −0.4 and just below −1.0 eV. This leads to the weak initial onset and a second stronger increase in the valence band DOS and experiment. In contrast, for MoSe_2_ and MoTe_2_, there is only one VB onset. In the band structures of MoSe_2_ and MoTe_2_, the uppermost valence bands are flatter than for MoS_2_, consistent with their steeper initial VB onset. Furthermore, there is a continuous density of states throughout the VB with no region of low density of states like that in MoS_2_. The values for the VBM, determined by the two different methods, are presented in Table 3. It can be seen that the values of the methods are consistent for both SXPS and HAXPES with the DOS fit method. Between the two methods, there is a slight disagreement which highlights the caveats discussed above with the extrapolation method as the VBM values differ between SXPS and HAXPES.

Laboratory-based XPS was utilized to measure the work function and ionization potentials of the materials. This was done by measuring and fitting the secondary electron cutoff edges seen in Figure S6. The work function values shown in Table 4 are obtained from the difference in energy between the photon energy and the secondary electron cutoff value. The WF values were found to be 5.30, 4.23, and 4.33 eV for MoS_2_, MoSe_2_, and MoTe_2_, respectively.

**Table 4:** Work Function and Ionization Potential Values for MoS_2_, MoSe_2_, and MoTe_2_ Obtained from XPS^a^

	WF (eV)	IP (eV)
MoS_2_	5.3	5.9
MoSe_2_	4.2	5.4
MoTe_2_	4.3	5.0

a The uncertainty is estimated to be ± 0.1 eV.

In the literature, the work function values for the MoX_2_ series vary considerably; see Table S3 in the SI for summary. The work function is the energy difference between the Fermi level and the vacuum level, and the Fermi-level position can differ for a multitude of reasons including growth method, surface contamination, and the number of layers. Starting with MoS_2_, which has the most reported WF values, the values range from 4.54 to 5.45 eV.^104−109^ MoSe_2_ has been reported to have a WF of 4.35 eV for MoSe_2_ fabricated by hot injection onto Bi_2_Se_3_,^110^ and 4.4 eV for an *in situ* exfoliated MoSe_2_ single crystal by Shimada et al.^107^ For MoTe_2_ a range of values from 3.8 to 4.35 eV has been reported.^107,111,112^ The WF values found in this study are in line with the reported values found in the literature.

A value that should be consistent across the literature is the ionization potential, which is the energy from the VBM to the vacuum level. This value, which can be seen in the second column of Table 4, is found by combining the work function value with the valence band edge to Fermi-level separation. The literature values for the ionization potential can also be found in SI Table S3. For MoS_2_, the literature values for the IP are 5.77^108^ and 5.60 eV^107^ for CVD-grown MoS_2_ on SiO_2_ and a single crystal, respectively. These values are in line with the value of 5.86 eV found in this work when experimental uncertainty is taken into account. Looking at MoSe_2_, the IP value of 5.40 eV also agrees with literature values of 5.34,^108^ 5.42,^107^ and 5.5 eV.^110^ Finally, for MoTe_2_, the value of 5.00 eV agrees with literature values of 5.0^107^ and 4.95 eV.^111^

**Figure 5:**
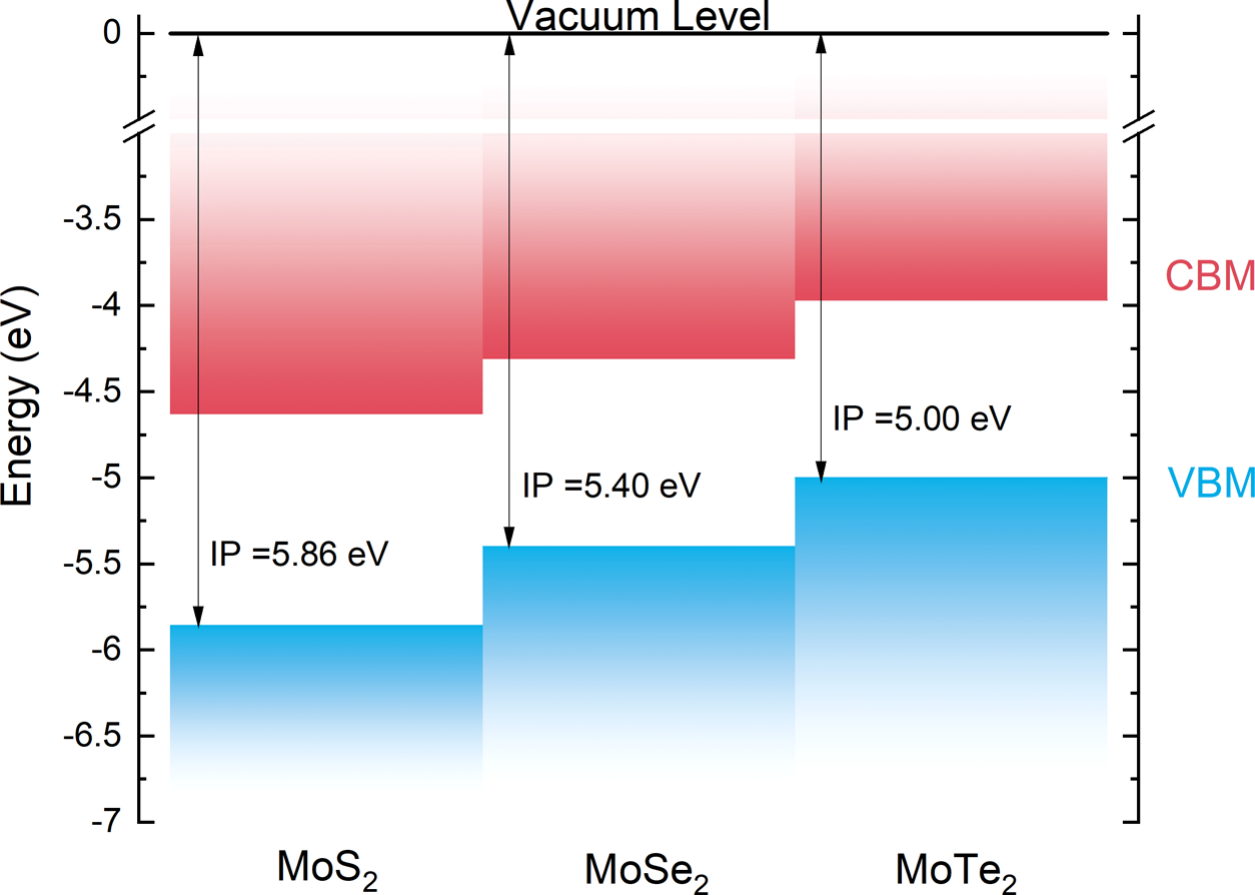
Band alignment between the three bulk dichalcogenides determined from the experimental ionization potentials and literature band gaps. The VBM and CBM are “pushed” to higher energy due to the energy of the chalcogen p orbitals becoming closer to that of the Mo 4d orbital.

Combining the IP values with literature band gap values,^113,114^ the band edges can be plotted with respect to the vacuum level, depicted in Figure 5. Both the ionization potential and electron affinity decrease from S → Te. This agrees with previously reported theoretical band alignments^34,108,115,116^ for both bulk and monolayer MoX_2_. These trends will be discussed in the next section.

## Comparison of Theoretical Density of States and Experimental Valence Band Spectra

Here, the calculated integrated density of states (DOS) is compared to the experimental valence band spectra. To do this, the theoretical DOS is photoionization cross-section-corrected and also broadened to account for lifetime broadening and instrumental resolution. One-electron photoionization cross sections interpolated from the calculations of Scofield^75^ were used to correct the calculated partial DOS. Then, Gaussian broadening of full width at half-maximum values of 0.28 and 0.26 eV was applied to account for instrumental broadening for the SXPS and HAXPES measurements, respectively. The Gaussian broadening was then fixed and Lorentzian broadening was applied accordingly until a reasonable fit was achieved. The Lorentzian broadening was ∼0.2 eV for all three dichalcogenides. The uncorrected PDOS can be seen in Figure S7 in the SI.

**Figure 6:**
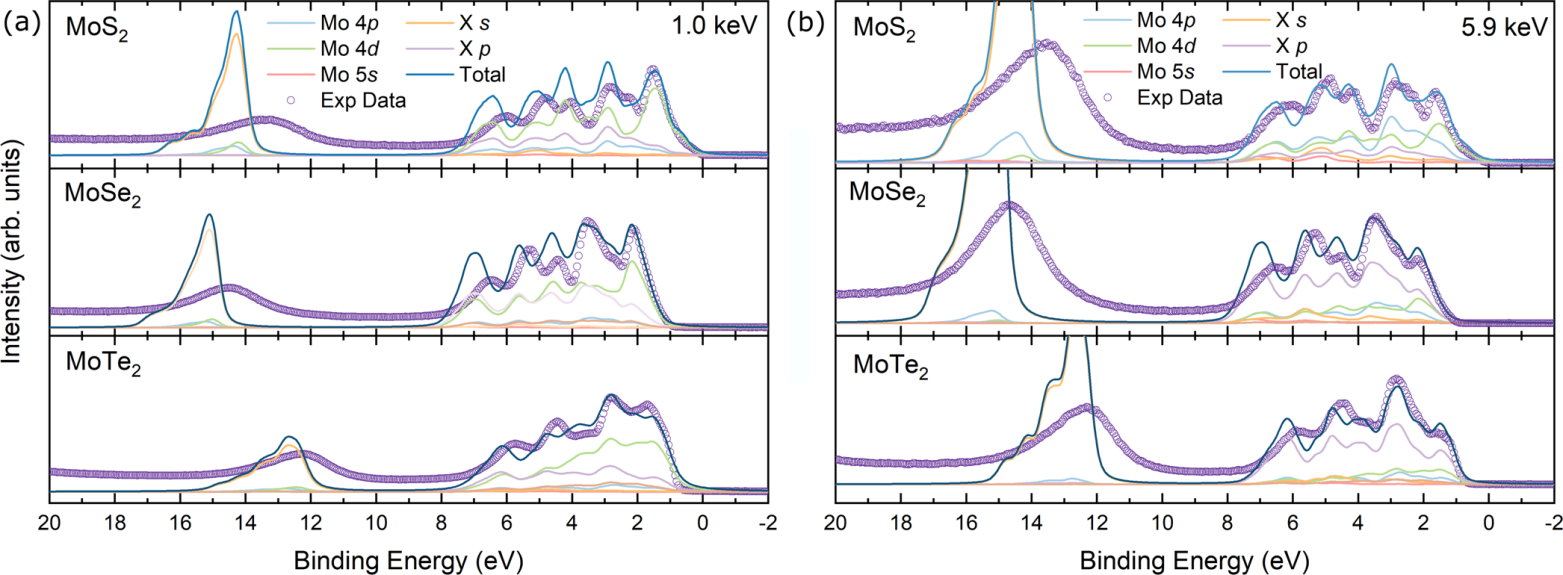
Broadened and cross-section-corrected theoretical density of states for MoS_2_, MoSe_2_, and MoTe_2_ compared with (a) the SXPS and (b) HAXPES valence band spectra.

The cross-section-corrected and broadened density of states is compared to the SXPS and HAXPES experimental valence band spectra in Figure 6a,b, respectively, for the three Mo-chalcogenides. For both SXPS and HAXPES valence bands, there is a reasonable agreement with the theory. For all spectra, the feature seen at ca. 12–15 eV, consisting of the chalcogen s orbitals alongside a small intensity from the Mo 5s, 4p and 4d, is seen to have an overestimated binding energy in the theory. This phenomenon has previously been attributed to photoemission final state effects not being included in the calculated density of states.^117^ Furthermore, semicore levels exhibit greater lifetime broadening relative to that of the valence states as they are more deeply bound. Therefore, the broadening applied to account for lifetime broadening is not sufficient at this energy and thus the experimental feature is much broader than the theory.^118^ Despite this, the feature can be seen to decrease in binding energy from MoS_2_ to MoTe_2_ due to the configuration energy of the chalcogen s decreasing from S to Te.^119^

The majority of the valence band is very similar for all three dichalcogenides with the main constituents being the Mo 4d and chalcogen p (S 3p, Se 4p, Te 5p) orbitals. As well as the two main orbital contributions to the valence band, there is also intensity from the chalcogen s and d (for Se and Te) as well as Mo 4p across most of the valence band. The significant contribution from the Mo 4d and chalcogen p is expected due to the covalent nature of the bonds between the transition metal and chalcogen that is exhibited by TMDs. What does change between the valence bands as you traverse down the series is the contributions at the valence band edge. Starting with MoS_2_, the valence band edge is seen to be strictly composed of the Mo 4d orbital, whereas MoSe_2_ and MoTe_2_ exhibit a mixture of the Mo 4d and chalcogen p orbitals. This is highlighted between the SXPS and HAXPES VB, where the Mo 4d orbital dominates the edge for SXPS, whereas for the HAXPES measurement the Se 4p/Te 5p orbital dominates. Furthermore, when the VBM is extracted for the SXPS and HAXPES measurements, they give the same value despite having different VB edge contributions for both MoSe_2_ and MoTe_2_.

The behavior of the Mo 4d and chalcogen p orbitals can be explained by the fact that, in the trigonal prismatic coordination of the transition metal, it is predicted by crystal field theory that the metal d orbital will split as discussed in the Introduction section. The nonbonding d_*z*^2^_ orbital is very evident when looking at the MoS_2_ data as the “bump” observed earlier consists of the d_*z*^2^_ orbital which is also known as the “nonbonding orbital”. However, as the anion is changed from S to Te, this feature is lost due to the increasing presence of the chalcogen p orbitals. This trend can be explained by looking at the configuration energies of the orbitals,^119,120^ plotted in Figure S8. The Te 5p orbital energy is 0.65 eV away from that of Mo 4d, whereas the S 3p orbital is 2.47 eV from the Mo 4d orbital, meaning that the overlap of the orbitals will be greater for MoTe_2_. The increased hybridization between the p and d orbitals explains the trends in the ionization potential in Figure 5, whereby the position of the chalcogen p level moving toward the vacuum level pushes the VBM closer to the vacuum level and in turn decreases the IP value down the series.

## Conclusions

A thorough investigation into the electronic structure of the molybdenum dichalcogenide series is presented. This is done through the utilization of hard and soft X-ray photoemission spectroscopy measurements coupled with density functional theory calculations. From the XPS core-level analysis, it was highlighted that when using higher photon energies the area ratio constraints used must be adjusted from the statistical value. This is due to the overlap of the initial doublet wavefunctions with the continuum wavefunction differing when the photon energy is increased. Furthermore, when increasing the photon energy, the relative photoionization cross section also changes, resulting in increased relative intensity of certain orbitals. This was pivotal in the determination of the Se 3s core-level binding energy with respect to that of the Mo 3d_5/2_ for MoSe_2_ as the Se 3s line is often overlooked in the literature. The binding energy was found to be 0.7 eV above the Mo 3d_5/2_ peak; this value should be used for any future XPS core-level analysis for MoSe_2_. As well as the core-level analysis, XPS also enabled the ionization potential values to be determined are were found to be 5.86, 5.40, and 5.00 eV for MoS_2_, MoSe_2_, and MoTe_2_, respectively. These values allowed for the determination of the band alignment of the three chalcogenides. The trend in the experimentally determined band alignment agreed well with previously calculated band positions. Finally, the experimental valence band was compared to cross-section-corrected and broadened theoretical density of states. Here, the orbital contributions were investigated and the role of p-d hybridization was seen to explain the trend in the properties of the materials. It was deduced that the strength of the p-d hybridization increased when traversing from the sulfide to the telluride due to a greater presence of chalcogen p orbitals at the valence band edge. This was explained by the configuration energy of the chalcogen p orbitals encroaching that of the Mo 4d from S to Te resulting in the increased mixing of these orbitals. Thus, this shifts the valence band maximum closer to the vacuum level which in turn decreases the ionization potential value.

In summary, this work provides an experimental account of the electronic structure which enhances the findings from previous theoretical studies. The experimentally determined band positions will be valuable to instruct future heterostructure-based devices. Furthermore, the ionization potential and work function values of the Mo dichalcogenides also give insight into the interfacial properties, such as the Schottky barrier height when in contact with metals or the alignment type when in contact with other semiconductors.

## Supporting Information Available

Area ratios used to fit core-level doublets at different photon energies, XPS spectra of as-entered and exfoliated MoS_2_, VBM positions from the linear extrapolation method, comparison of the DFT band structure with the valence band spectrum of MoS_2_, valence band and secondary electron cutoff XPS measurements from MoS_2_, MoSe_2_, and MoTe_2_, literature values of work functions and ionization potentials, and orbital configuration energies for Mo, S, Se, and Te (PDF)

## References

[ref1] Qian Z.; Jiao L.; Xie L. Phase Engineering of Two-Dimensional Transition Metal Dichalcogenides. Chin. J. Chem. 2020, 38, 753–760. 10.1002/cjoc.202000064.

[ref2] Eftekhari A. Molybdenum diselenide MoSe_2_ for energy storage, catalysis, and optoelectronics. Appl. Mater. Today 2017, 8, 1–17. 10.1016/j.apmt.2017.01.006.

[ref3] Santosh S. K.; Zhang C.; Hong S.; Wallace R. M.; Cho K. Phase stability of transition metal dichalcogenide by competing ligand field stabilization and charge density wave. 2D Mater. 2015, 2, 035019 10.1088/2053-1583/2/3/035019.

[ref4] Jin Q.; Liu N.; Chen B.; Mei D. Mechanisms of Semiconducting 2H to Metallic 1T Phase Transition in Two-dimensional MoS_2_ Nanosheets. J. Phys. Chem. C 2018, 122, 28215–28224. 10.1021/acs.jpcc.8b10256.

[ref5] Hanson E. D.; Lilley L. M.; Cain J. D.; Hao S.; Palacios E.; Aydin K.; Wolverton C.; Meade T.; Dravid V. P. Phase engineering and optical properties of 2D MoSe_2_: Promise and pitfalls. Mater. Chem. Phys. 2019, 225, 219–226. 10.1016/j.matchemphys.2018.11.069.

[ref6] Deng Y.; Zhao X.; Zhu C.; Li P.; Duan R.; Liu G.; Liu Z. MoTe_2_: Semiconductor or Semimetal?. ACS Nano 2021, 15, 12465–12474. 10.1021/acsnano.1c01816. 34379388

[ref7] Momma K.; Izumi F. *VESTA3* for three-dimensional visualization of crystal, volumetric and morphology data. J. Appl. Crystallogr. 2011, 44, 1272–1276. 10.1107/S0021889811038970.

[ref8] Choudhary N.; Patel M.; Ho Y.-H.; Dahotre N. B.; Lee W.; Hwang J. Y.; Choi W. Directly deposited MoS_2_ thin film electrodes for high performance supercapacitors. J. Mater. Chem. A 2015, 3, 24049–24054. 10.1039/C5TA08095A.

[ref9] Zou J.; Li F.; Bissett M. A.; Kim F.; Hardwick L. J. Intercalation behaviour of Li and Na into 3-layer and multilayer MoS_2_ flakes. Electrochim. Acta 2020, 331, 135284 10.1016/j.electacta.2019.135284.

[ref10] Panda M. R.; Gangwar R.; Muthuraj D.; Sau S.; Pandey D.; Banerjee A.; Chakrabarti A.; Sagdeo A.; Weyland M.; Majumder M.; et al. High Performance Lithium-Ion Batteries Using Layered 2H-MoTe_2_ as Anode. Small 2020, 16, 2002669 10.1002/smll.202002669. 32803832

[ref11] Wu Y.-C.; Liu W.-R. Few-layered MoSe_2_ ultrathin nanosheets as anode materials for lithium ion batteries. J. Alloys Compd. 2020, 813, 152074 10.1016/j.jallcom.2019.152074.

[ref12] Sarkar D.; Liu W.; Xie X.; Anselmo A. C.; Mitragotri S.; Banerjee K. MoS_2_ Field-Effect Transistor for Next-Generation Label-Free Biosensors. ACS Nano 2014, 8, 3992–4003. 10.1021/nn5009148. 24588742

[ref13] Late D. J.; Huang Y.-K.; Liu B.; Acharya J.; Shirodkar S. N.; Luo J.; Yan A.; Charles D.; Waghmare U. V.; Dravid V. P.; Rao C. N. R. Sensing Behavior of Atomically Thin-Layered MoS_2_ Transistors. ACS Nano 2013, 7, 4879–4891. 10.1021/nn400026u. 23713986

[ref14] He Q.; Zeng Z.; Yin Z.; Li H.; Wu S.; Huang X.; Zhang H. Fabrication of Flexible MoS_2_ Thin-Film Transistor Arrays for Practical Gas-Sensing Applications. Small 2012, 8, 2994–2999. 10.1002/smll.201201224. 22778003

[ref15] Li H.; Yin Z.; He Q.; Li H.; Huang X.; Lu G.; Fam D. W. H.; Tok A. I. Y.; Zhang Q.; Zhang H. Fabrication of Single- and Multilayer MoS_2_ Film-Based Field-Effect Transistors for Sensing NO at Room Temperature. Small 2012, 8, 63–67. 10.1002/smll.201101016. 22012880

[ref16] Singh S.; Deb J.; Sarkar U.; Sharma S. MoSe_2_ Crystalline Nanosheets for Room-Temperature Ammonia Sensing. ACS Appl. Nano Mater. 2020, 3, 9375–9384. 10.1021/acsanm.0c02011.

[ref17] Radisavljevic B.; Radenovic A.; Brivio J.; Giacometti V.; Kis A. Single-layer MoS_2_ transistors. Nat. Nanotechnol. 2011, 6, 147–150. 10.1038/nnano.2010.279. 21278752

[ref18] Gao L. Flexible Device Applications of 2D Semiconductors. Small 2017, 13, 1603994 10.1002/smll.201603994. 28464480

[ref19] Ornelas C. D.; Bowman A.; Walmsley T. S.; Wang T.; Andrews K.; Zhou Z.; Xu Y.-Q. Ultrafast Photocurrent Response and High Detectivity in Two-Dimensional MoSe_2_-based Heterojunctions. ACS Appl. Mater. Interfaces 2020, 12, 46476–46482. 10.1021/acsami.0c12155. 32867473

[ref20] Kang S.; Lee D.; Kim J.; Capasso A.; Kang H. S.; Park J.-W.; Lee C.-H.; Lee G.-H. 2D semiconducting materials for electronic and optoelectronic applications: potential and challenge. 2D Mater. 2020, 7, 022003 10.1088/2053-1583/ab6267.

[ref21] Timpel M.; Ligorio G.; Ghiami A.; Gavioli L.; Cavaliere E.; Chiappini A.; Rossi F.; Pasquali L.; Gärisch F.; List-Kratochvil E. J. W.; et al. 2D-MoS_2_ goes 3D: transferring optoelectronic properties of 2D MoS_2_ to a large-area thin film. npj 2D Mater. Appl. 2021, 5, 64 10.1038/s41699-021-00244-x.

[ref22] Pospischil A.; Furchi M. M.; Mueller T. Solar-energy conversion and light emission in an atomic monolayer p-n diode. Nat. Nanotechnol. 2014, 9, 257 10.1038/nnano.2014.14. 24608229

[ref23] Lopez-Sanchez O.; Lembke D.; Kayci M.; Radenovic A.; Kis A. Ultrasensitive photodetectors based on monolayer MoS_2_. Nat. Nanotechnol. 2013, 8, 497 10.1038/nnano.2013.100. 23748194

[ref24] Taffelli A.; Diré S.; Quaranta A.; Pancheri L. MoS_2_ Based Photodetectors: A Review. Sensors 2021, 21, 2758 10.3390/s21082758. 33919731 PMC8070690

[ref25] Ross J. S.; Klement P.; Jones A. M.; Ghimire N. J.; Yan J.; Mandrus D. G.; Taniguchi T.; Watanabe K.; Kitamura K.; Yao W.; et al. Electrically tunable excitonic light-emitting diodes based on monolayer WSe_2_ p-n junctions. Nat. Nanotechnol. 2014, 9, 268–272. 10.1038/nnano.2014.26. 24608230

[ref26] Amit I.; Octon T. J.; Townsend N. J.; Reale F.; Wright C. D.; Mattevi C.; Craciun M. F.; Russo S. Role of Charge Traps in the Performance of Atomically Thin Transistors. Adv. Mater. 2017, 29, 1605598 10.1002/adma.201605598. 28295639

[ref27] Kim B. S.; Kyung W. S.; Seo J. J.; Kwon J. Y.; Denlinger J. D.; Kim C.; Park S. R. Possible electric field induced indirect to direct band gap transition in MoSe_2_. Sci. Rep. 2017, 7, 5206 10.1038/s41598-017-05613-5. 28701785 PMC5507882

[ref28] Hossain M. A.; Merzougui B. A.; Alharbi F. H.; Tabet N. Electrochemical deposition of bulk MoS_2_ thin films for photovoltaic applications. Sol. Energy Mater. Sol. Cells 2018, 186, 165–174. 10.1016/j.solmat.2018.06.026.

[ref29] Kim E.-K.; Yoon S. J.; Bui H. T.; Patil S. A.; Bathula C.; Shrestha N. K.; Im H.; Han S.-H. Epitaxial electrodeposition of single crystal MoTe_2_ nanorods and Li+ storage feasibility. J. Electroanal. Chem. 2020, 878, 114672 10.1016/j.jelechem.2020.114672.

[ref30] Shackery I.; Pezeshki A.; Park J. Y.; Palanivel U.; Kwon H. J.; Yoon H. S.; Im S.; Cho J. S.; Jun S. C. Few-layered α-MoTe_2_ Schottky junction for a high sensitivity chemical-vapour sensor. J. Mater. Chem. C 2018, 6, 10714–10722. 10.1039/C8TC02635A.

[ref31] Wu E.; Xie Y.; Yuan B.; Zhang H.; Hu X.; Liu J.; Zhang D. Ultrasensitive and Fully Reversible NO_2_ Gas Sensing Based on p-Type MoTe_2_ under Ultraviolet Illumination. ACS Sens. 2018, 3, 1719–1726. 10.1021/acssensors.8b00461. 30105902

[ref32] Octon T. J.; Nagareddy V. K.; Russo S.; Craciun M. F.; Wright C. D. Fast High-Responsivity Few-Layer MoTe_2_ Photodetectors. Adv. Opt. Mater. 2016, 4, 1750–1754. 10.1002/adom.201600290.

[ref33] Bie Y.-Q.; Grosso G.; Heuck M.; Furchi M. M.; Cao Y.; Zheng J.; Bunandar D.; Navarro-Moratalla E.; Zhou L.; Efetov D. K.; et al. A MoTe_2_-based light-emitting diode and photodetector for silicon photonic integrated circuits. Nat. Nanotechnol. 2017, 12, 1124–1129. 10.1038/nnano.2017.209. 29209014

[ref34] Jiang H. Electronic Band Structures of Molybdenum and Tungsten Dichalcogenides by the GW Approach. J. Phys. Chem. C 2012, 116, 7664–7671. 10.1021/jp300079d.

[ref35] Chen X.; Chen Z.; Li J. Critical electronic structures controlling phase transitions induced by lithium ion intercalation in molybdenum disulphide. Chin. Sci. Bull. 2013, 58, 1632–1641. 10.1007/s11434-013-5834-y.

[ref36] Pike N. A.; Van Troeye B.; Dewandre A.; Petretto G.; Gonze X.; Rignanese G.-M.; Verstraete M. J. Origin of the counterintuitive dynamic charge in the transition metal dichalcogenides. Phys. Rev. B 2017, 95, 201106 10.1103/PhysRevB.95.201106.

[ref37] Rahman I. A.; Purqon A. First Principles Study of Molybdenum Disulfide Electronic Structure. J. Phys.: Conf. Ser. 2017, 877, 012026 10.1088/1742-6596/877/1/012026.

[ref38] Böker T.; Severin R.; Muller A.; Janowitz C.; Manzke R.; D V.; Kruger P.; Mazur A.; Pollmann J. Band structure of MoS_2_, MoSe_2_, and α-MoTe_2_: Angle-resolved photoelectron spectroscopy and ab initio calculations. Phys. Rev. B 2001, 64, 235305 10.1103/PhysRevB.64.235305.

[ref39] Lin X.; Li W.; Dong Y.; Wang C.; Chen Q.; Zhang H. Two-dimensional metallic MoS_2_: A DFT study. Comput. Mater. Sci. 2016, 124, 49–53. 10.1016/j.commatsci.2016.07.020.

[ref40] Gusakova J.; Wang X.; Shiau L. L.; Krivosheeva A.; Shaposhnikov V.; Borisenko V.; Gusakov V.; Tay B. K. Electronic Properties of Bulk and Monolayer TMDs: Theoretical Study Within DFT Framework (GVJ-2e Method). Phys. Status Solidi A 2017, 214, 1700218 10.1002/pssa.201700218.

[ref41] Coehoorn R.; Haas C.; Dijkstra J.; Flipse C. J. F.; de Groot R. A.; Wold A. Electronic structure of MoSe_2_, MoS_2_, and WSe_2_. I. Band-structure calculations and photoelectron spectroscopy. Phys. Rev. B 1987, 35, 6195–6202. 10.1103/PhysRevB.35.6195. 9940850

[ref42] Lee T.-L.; Duncan D. A. A Two-Color Beamline for Electron Spectroscopies at Diamond Light Source. Synchrotron Radiat. News 2018, 31, 16–22. 10.1080/08940886.2018.1483653.

[ref43] Kresse G.; Hafner J. *Ab initio* molecular dynamics for liquid metals. Phys. Rev. B 1993, 47, 558–561. 10.1103/PhysRevB.47.558. 10004490

[ref44] Kresse G.; Hafner J. Ab initio molecular-dynamics simulation of the liquid-metal amorphous-semiconductor transition in germanium. Phys. Rev. B 1994, 49, 14251–14269. 10.1103/PhysRevB.49.14251. 10010505

[ref45] Kresse G.; Furthmüller J. Efficient iterative schemes for ab initio total-energy calculations using a plane-wave basis set. Phys. Rev. B 1996, 54, 11169–11186. 10.1103/PhysRevB.54.11169. 9984901

[ref46] Kresse G.; Furthmüller J. Efficiency of ab initio total energy calculations for metals and semiconductors using a plane wave basis set. Comput. Mater. Sci. 1996, 6, 15 10.1016/0927-0256(96)00008-0. 9984901

[ref47] Blöchl P. E. Projector augmented-wave method. Phys. Rev. B 1994, 50, 17953–17979. 10.1103/PhysRevB.50.17953. 9976227

[ref48] Hellenbrandt M. The Inorganic Crystal Structure Database (ICSD)—Present and Future. Crystallogr. Rev. 2004, 10, 17–22. 10.1080/08893110410001664882.

[ref49] Takeuchi Y.; Nowacki W. Detailed crystal structure of rhombohedral MoS_2_ and systematic deduction of possible polytypes of molybdenite. Schweiz. Mineral. Petrogr. Mitt. 1964, 44, 105–120.

[ref50] Dickinson R. G.; Pauling L. The Crystal Structure of Molybdenute. J. Am. Chem. Soc. 1923, 45, 1466–1471. 10.1021/ja01659a020.

[ref51] Bronsema K. D.; Boer J. L. D.; Jellinek F. On the structure of molybdenum diselenide and disulfide. Z. Anorg. Allg. Chem. 1986, 540, 15–17. 10.1002/zaac.19865400904.

[ref52] Puotinen D.; Newnham R. E. The crystal structure of MoTe_2_. Acta Crystallogr. 1961, 14, 691–692. 10.1107/S0365110X61002084.

[ref53] Perdew J. P.; Burke K.; Ernzerhof M. Generalized Gradient Approximation Made Simple. Phys. Rev. Lett. 1996, 77, 3865–3868. 10.1103/PhysRevLett.77.3865. 10062328

[ref54] Grimme S.; Ehrlich S.; Goerigk L. Effect of the damping function in dispersion corrected density functional theory. J. Comput. Chem. 2011, 32, 1456–1465. 10.1002/jcc.21759. 21370243

[ref55] Heyd J.; Scuseria G. E.; Ernzerhof M. Hybrid functionals based on a screened Coulomb potential. J. Chem. Phys. 2003, 118, 8207–8215. 10.1063/1.1564060.

[ref56] Heyd J.; Scuseria G. E.; Ernzerhof M. Erratum: “Hybrid functionals based on a screened Coulomb potential” [J. Chem. Phys. 118, 8207 (2003)]. J. Chem. Phys. 2006, 124, 219906 10.1063/1.2204597.

[ref57] Ganose A.; Jackson A. J.; Scanlon D. O. sumo: Command-line tools for plotting and analysis of periodic ab initio calculations. J. Open Source Softw. 2018, 3, 717 10.21105/joss.00717.

[ref58] Fan J.-H.; Gao P.; Zhang A.-M.; Zhu B.-R.; Zeng H.-L.; Cui X.-D.; He R.; Zhang Q.-M. Resonance Raman scattering in bulk 2H-MX_2_ (M = Mo, W; X = S, Se) and monolayer MoS_2_. J. Appl. Phys. 2014, 115, 053527 10.1063/1.4862859.

[ref59] Sekine T.; Izumi M.; Nakashizu T.; Uchinokura K.; Matsuura E. Raman Scattering and Infrared Reflectance in 2H-MoSe_2_. J. Phys. Soc. Jpn. 1980, 49, 1069–1077. 10.1143/JPSJ.49.1069.

[ref60] Liang L.; Meunier V. First-principles Raman spectra of MoS_2_, WS_2_ and their heterostructures. Nanoscale 2014, 6, 5394–5401. 10.1039/c3nr06906k. 24710269

[ref61] Li H.; Zhang Q.; Yap C. C. R.; Tay B. K.; Edwin T. H. T.; Olivier A.; Baillargeat D. From Bulk to Monolayer MoS_2_: Evolution of Raman Scattering. Adv. Funct. Mater. 2012, 22, 1385–1390. 10.1002/adfm.201102111.

[ref62] Yamamoto M.; Wang S. T.; Ni M.; Lin Y.-F.; Li S.-L.; Aikawa S.; Jian W.-B.; Ueno K.; Wakabayashi K.; Tsukagoshi K. Strong Enhancement of Raman Scattering from a Bulk-Inactive Vibrational Mode in Few-Layer MoTe_2_. ACS Nano 2014, 8, 3895–3903. 10.1021/nn5007607. 24654654

[ref63] Grzeszczyk M.; Gołasa K.; Zinkiewicz M.; Nogajewski K.; Molas M. R.; Potemski M.; Wysmołek A.; Babiński A. Raman scattering of few-layers MoTe_2_. 2D Mater. 2016, 3, 025010 10.1088/2053-1583/3/2/025010. PMC6288152 30531971

[ref64] Cheng S.; Yang L.; Li J.; Liu Z.; Zhang W.; Chang H. Large area, phase-controlled growth of few-layer, two-dimensional MoTe_2_ and lateral 1T’-2H heterostructures by chemical vapor deposition. CrystEngComm 2017, 19, 1045–1051. 10.1039/C6CE02506D.

[ref65] Camacho-López M.; Escobar-Alarcón L.; Picquart M.; Arroyo R.; Córdoba G.; Haro-Poniatowski E. Micro-Raman study of the m-MoO_2_ to α-MoO_3_ transformation induced by cw-laser irradiation. Opt. Mater. 2011, 33, 480–484. 10.1016/j.optmat.2010.10.028.

[ref66] Mahatha S. K.; Patel K. D.; Menon K. S. R. Electronic structure investigation of MoS_2_ and MoSe_2_ using angle-resolved photoemission spectroscopy and ab initio band structure studies. J. Phys.: Condens. Matter 2012, 24, 475–504. 10.1088/0953-8984/24/47/475504. 23110779

[ref67] James P. B.; Lavik M. T. The crystal structure of MoSe_2_. Acta Crystallogr. 1963, 16, 1183 10.1107/S0365110X6300311X.

[ref68] Agarwal M. K.; Capers M. J. The measurement of the lattice parameters of molybdenum ditelluride. J. Appl. Crystallogr. 1972, 5, 63–66. 10.1107/S0021889872008787.

[ref69] Zelewski S. J.; Kudrawiec R. Photoacoustic and modulated reflectance studies of indirect and direct band gap in van der Waals crystals. Sci. Rep. 2017, 7, 15365 10.1038/s41598-017-15763-1. 29133933 PMC5684221

[ref70] Kopaczek J.; Zelewski S. J.; Polak M. P.; Gawlik A.; Chiappe D.; Schulze A.; Caymax M.; Kudrawiec R. Direct and indirect optical transitions in bulk and atomically thin MoS_2_ studied by photoreflectance and photoacoustic spectroscopy. J. Appl. Phys. 2019, 125, 135701 10.1063/1.5080300.

[ref71] Choi B. K.; Kim M.; Jung K.-H.; Kim J.; Yu K.-S.; Chang Y. J. Temperature dependence of band gap in MoSe_2_ grown by molecular beam epitaxy. Nanoscale Res. Lett. 2017, 12, 492 10.1186/s11671-017-2266-7. 28812234 PMC5557720

[ref72] Lezama I. G.; Arora A.; Ubaldini A.; Barreteau C.; Giannini E.; Potemski M.; Morpurgo A. F. Indirect-to-Direct Band Gap Crossover in Few-Layer MoTe_2_. Nano Lett. 2015, 15, 2336–2342. 10.1021/nl5045007. 25803208

[ref73] Goldberg A. M.; Beal A. R.; Lévy F. A.; Davis E. A. The low-energy absorption edge in 2H-MoS_2_ and 2H-MoSe_2_. Philos. Mag. 1975, 32, 367–378. 10.1080/14786437508219961.

[ref74] Hu S.; Lee Y.; Shen J.; Chen K.; Tiong K.; Huang Y. Temperature dependence of absorption edge anisotropy in 2H-MoSe_2_ layered semiconductors. Solid State Commun. 2006, 139, 176–180. 10.1016/j.ssc.2006.05.027.

[ref75] Scofield J. H. Theoretical Photoionization Cross Sections from 1 to 1500 keV, UCRL–51326; California Univ., Lawrence Livermore Lab: Livermore, 1973; p 376.

[ref76] Walker T. E. H.; Berkowitz J.; Dehmer J. L.; Waber J. T. Nonstatistical Ratios of Photoionization Cross Sections for States Split by Spin-Orbit Coupling. Phys. Rev. Lett. 1973, 31, 678–681. 10.1103/PhysRevLett.31.678.

[ref77] Coster D.; Kronig R. D. L. New type of Auger effect and its influence on the x-ray spectrum. Physica 1935, 2, 13–24. 10.1016/S0031-8914(35)90060-X.

[ref78] Major G. H.; Fairley N.; Sherwood P. M. A.; Linford M. R.; Terry J.; Fernandez V.; Artyushkova K. Practical guide for curve fitting in x-ray photoelectron spectroscopy. J. Vac. Sci. Technol. A 2020, 38, 061203 10.1116/6.0000377.

[ref79] Nolot E.; Cadot S.; Martin F.; Hönicke P.; Zech C.; Beckhoff B. In-line characterization of ultrathin transition metal dichalcogenides using X-ray fluorescence and X-ray photoelectron spectroscopy. Spectrochim. Acta, Part B 2020, 166, 105788 10.1016/j.sab.2020.105788.

[ref80] Mårtensson N.; Nyholm R. Electron spectroscopic determinations of M and N core-hole lifetimes for the elements Nb–Te (Z = 41 - 52). Phys. Rev. B 1981, 24, 7121–7134. 10.1103/PhysRevB.24.7121.

[ref81] Qin P.; Fang G.; Ke W.; Cheng F.; Zheng Q.; Wan J.; Lei H.; Zhao X. In situ growth of double-layer MoO_3_/MoS_2_ film from MoS_2_ for hole-transport layers in organic solar cell. J. Mater. Chem. A 2014, 2, 2742–2756. 10.1039/c3ta13579a.

[ref82] Mawlong L. P. L.; Bora A.; Giri P. K. Coupled Charge Transfer Dynamics and Photoluminescence Quenching in Monolayer MoS_2_ Decorated with WS_2_ Quantum Dots. Sci. Rep. 2019, 9, 19414 10.1038/s41598-019-55776-6. 31857608 PMC6923361

[ref83] Ho Y.-T.; Ma C.-H.; Luong T.-T.; Wei L.-L.; Yen T.-C.; Hsu W.-T.; Chang W.-H.; Chu Y.-C.; Tu Y.-Y.; Pande K. P.; Chang E. Y. Layered MoS_2_ grown on c-sapphire by pulsed laser deposition. Phys. Status Solidi RRL 2015, 9, 187–191. 10.1002/pssr.201409561.

[ref84] Cai Y.; Yang X.; Liang T.; Dai L.; Ma L.; Huang G.; Chen W.; Chen H.; Su H.; Xu M. Easy incorporation of single-walled carbon nanotubes into two-dimensional MoS_2_ for high-performance hydrogen evolution. Nanotechnology 2014, 25, 465401 10.1088/0957-4484/25/46/465401. 25360803

[ref85] Kim S. K.; Song W.; Ji S.; Lim Y. R.; Lee Y. B.; Myung S.; Lim J.; An K.-S.; Lee S. S. Synergetic effect at the interfaces of solution processed MoS_2_-WS_2_ composite for hydrogen evolution reaction. Appl. Surf. Sci. 2017, 425, 241–245. 10.1016/j.apsusc.2017.06.211.

[ref86] Yang W.; Sun Q.-Q.; Geng Y.; Chen L.; Zhou P.; Ding S.-J.; Zhang D. W. The Integration of Sub-10 nm Gate Oxide on MoS_2_ with Ultra Low Leakage and Enhanced Mobility. Sci. Rep. 2015, 5, 11921 10.1038/srep11921. 26146017 PMC4491716

[ref87] Kondekar N. P.; Boebinger M. G.; Woods E. V.; McDowell M. T. In Situ XPS Investigation of Transformations at Crystallographically Oriented MoS_2_ Interfaces. ACS Appl. Mater. Interfaces 2017, 9, 32394–32404. 10.1021/acsami.7b10230. 28846377

[ref88] Choi J.-G.; Thompson L. XPS study of as-prepared and reduced molybdenum oxides. Appl. Surf. Sci. 1996, 93, 143–149. 10.1016/0169-4332(95)00317-7.

[ref89] Ganta D.; Sinha S.; Haasch R. T. 2-D Material Molybdenum Disulfide Analyzed by XPS. Surf. Sci. Spectra 2014, 21, 19–27. 10.1116/11.20140401.

[ref90] Bana H.; Travaglia E.; Bignardi L.; et al. Epitaxial growth of single-orientation high-quality MoS_2_ monolayers. 2D Mater. 2018, 5, 035012 10.1088/2053-1583/aabb74.

[ref91] Silva C. C.; Dombrowski D.; Atodiresei N.; Jolie W.; zum Hagen F. F.; Cai J.; Ryan P. T. P.; Thakur P. K.; Caciuc V.; Blügel S.; et al. Spatial variation of geometry, binding, and electronic properties in the moiré superstructure of MoS_2_ on Au(111). 2D Mater. 2022, 9, 025003 10.1088/2053-1583/ac4958.

[ref92] Kayanuma Y. Hard X-ray Photoelectron Spectroscopy (HAXPES); Springer International Publishing: Cham, 2016; pp 175–195.

[ref93] Kalha C.; Fernando N. K.; Bhatt P.; et al. Hard x-ray photoelectron spectroscopy: a snapshot of the state-of-the-art in 2020. J. Phys.: Condens. Matter 2021, 33, 233001 10.1088/1361-648X/abeacd. 33647896

[ref94] Roy A.; Movva H. C. P.; Satpati B.; Kim K.; Dey R.; Rai A.; Pramanik T.; Guchhait S.; Tutuc E.; Banerjee S. K. Structural and Electrical Properties of MoTe_2_ and MoSe_2_ Grown by Molecular Beam Epitaxy. ACS Appl. Mater. Interfaces 2016, 8, 7396–7402. 10.1021/acsami.6b00961. 26939890

[ref95] Abdallah W. A.; Nelson A. E. Characterization of MoSe_2_(0001) and ion-sputtered MoSe_2_ by XPS. J. Mater. Sci. 2005, 40, 2679–2681. 10.1007/s10853-005-2104-7.

[ref96] Truong Q. D.; Kempaiah Devaraju M.; Nakayasu Y.; Tamura N.; Sasaki Y.; Tomai T.; Honma I. Exfoliated MoS_2_ and MoSe_2_ Nanosheets by a Supercritical Fluid Process for a Hybrid Mg-Li–Ion Battery. ACS Omega 2017, 2, 2360–2367. 10.1021/acsomega.7b00379. 31457585 PMC6640930

[ref97] Zhao Y.; Lee H.; Choi W.; Fei W.; Lee C. J. Large-area synthesis of monolayer MoSe_2_ films on SiO_2_/Si substrates by atmospheric pressure chemical vapor deposition. RSC Adv. 2017, 7, 27969–27973. 10.1039/C7RA03642F.

[ref98] Damien D.; Anil A.; Chatterjee D.; Shaijumon M. M. Direct deposition of MoSe_2_ nanocrystals onto conducting substrates: towards ultra-efficient electrocatalysts for hydrogen evolution. J. Mater. Chem. A 2017, 5, 13364–13372. 10.1039/C6TA09645J.

[ref99] Timoshkin A. N.; Sobolev V. V.; Sobolev V. V. Electron characteristic loss spectra of molybdenum dichalcogenides. Phys. Solid State 2000, 42, 37–40. 10.1134/1.1131164.

[ref100] Beal A. R.; Hughes H. P. Kramers-Kronig analysis of the reflectivity spectra of 2H-MoS_2_, 2H-MoSe_2_ and 2H-MoTe_2_. J. Phys. C: Solid State Phys. 1979, 12, 881–890. 10.1088/0022-3719/12/5/017.

[ref101] Zavabeti A.; Aukarasereenont P.; Tuohey H.; et al. High-mobility p-type semiconducting two-dimensional β-TeO_2_. Nat. Electron. 2021, 4, 277–283. 10.1038/s41928-021-00561-5.

[ref102] Bahl M. K.; Watson R. L.; Irgolic K. J. X-ray photoemission studies of tellurium and some of its compounds. J. Chem. Phys. 1977, 66, 5526–5535. 10.1063/1.433874.

[ref103] Swallow J. E. N.; Varley J. B.; Jones L. A. H.; Gibbon J. T.; Piper L. F. J.; Dhanak V. R.; Veal T. D. Transition from electron accumulation to depletion at β-Ga_2_O_3_ surfaces: The role of hydrogen and the charge neutrality level. APL Mater. 2019, 7, 022528 10.1063/1.5054091.

[ref104] Lin J.; Zhong J.; Zhong S.; Li H.; Zhang H.; Chen W. Modulating electronic transport properties of MoS_2_ field effect transistor by surface overlayers. Appl. Phys. Lett. 2013, 103, 063109 10.1063/1.4818463.

[ref105] Diaz H. C.; Ma Y.; Chaghi R.; Batzill M. High density of (pseudo) periodic twin-grain boundaries in molecular beam epitaxy-grown van der Waals heterostructure: MoTe_2_/MoS_2_. Appl. Phys. Lett. 2016, 108, 191606 10.1063/1.4949559.

[ref106] Lee S. Y.; Kim U. J.; Chung J.; Nam H.; Jeong H. Y.; Han G. H.; Kim H.; Oh H. M.; Lee H.; Kim H.; et al. Large Work Function Modulation of Monolayer MoS_2_ by Ambient Gases. ACS Nano 2016, 10, 6100–6107. 10.1021/acsnano.6b01742. 27232340

[ref107] Shimada T.; Ohuchi F. S.; Parkinson B. A. Work Function and Photothreshold of Layered Metal Dichalcogenides. Jpn. J. Appl. Phys. 1994, 33, 2696–2698. 10.1143/JJAP.33.2696.

[ref108] Keyshar K.; Berg M.; Zhang X.; Vajtai R.; Gupta G.; Chan C. K.; Beechem T. E.; Ajayan P. M.; Mohite A. D.; Ohta T. Experimental Determination of the Ionization Energies of MoSe_2_, WS_2_, and MoS_2_ on SiO_2_ Using Photoemission Electron Microscopy. ACS Nano 2017, 11, 8223–8230. 10.1021/acsnano.7b03242. 28723073

[ref109] Schlaf R.; Lang O.; Pettenkofer C.; Jaegermann W. Band lineup of layered semiconductor heterointerfaces prepared by van der Waals epitaxy: Charge transfer correction term for the electron affinity rule. J. Appl. Phys. 1999, 85, 2732–2753. 10.1063/1.369590.

[ref110] Yang J.; Wang C.; Ju H.; Sun Y.; Xing S.; Zhu J.; Yang Q. Integrated Quasiplane Heteronanostructures of MoSe_2_/Bi_2_Se_3_ Hexagonal Nanosheets: Synergetic Electrocatalytic Water Splitting and Enhanced Supercapacitor Performance. Adv. Funct. Mater. 2017, 27, 1703864 10.1002/adfm.201703864.

[ref111] Williams R. H. The structure of the upper valence bands in MoTe_2_ and NbSe_2_. J. Phys. C: Solid State Phys. 1973, 6, L32–L35. 10.1088/0022-3719/6/1/010.

[ref112] Schlaf R.; Tiefenbacher S.; Lang O.; Pettenkofer C.; Jaegermann W. Van der Waals epitaxy of thin InSe films on MoTe_2_. Surf. Sci. 1994, 303, L343–L347. 10.1016/0039-6028(94)90610-6.

[ref113] Conan A.; Delaunay D.; Bonnet A.; Moustafa A. G.; Spiesser M. Temperature dependence of the electrical conductivity and thermoelectric power in MoTe_2_ single crystals. Phys. Status Solidi B 1979, 94, 279–286. 10.1002/pssb.2220940132.

[ref114] Kam K. K.; Parkinson B. A. Detailed photocurrent spectroscopy of the semiconducting group VIB transition metal dichalcogenides. J. Phys. Chem. A 1982, 86, 463–467. 10.1021/j100393a010.

[ref115] Liu Y.; Stradins P.; Wei S.-H. Van der Waals metal-semiconductor junction: Weak Fermi level pinning enables effective tuning of Schottky barrier. Sci. Adv. 2016, 2, e1600069 10.1126/sciadv.1600069. 27152360 PMC4846439

[ref116] Guo Y.; Robertson J. Band engineering in transition metal dichalcogenides: Stacked versus lateral heterostructures. Appl. Phys. Lett. 2016, 108, 233104 10.1063/1.4953169.

[ref117] Ley L.; Pollak R. A.; McFeely F. R.; Kowalczyk S. P.; Shirley D. A. Total valence-band densities of states of III-V and II-VI compounds from x-ray photoemission spectroscopy. Phys. Rev. B 1974, 9, 600–621. 10.1103/PhysRevB.9.600.

[ref118] Swallow J. E. N.; Vorwerk C.; Mazzolini P.; et al. Influence of Polymorphism on the Electronic Structure of Ga_2_O_3_. Chem. Mater. 2020, 32, 8460–8470. 10.1021/acs.chemmater.0c02465.

[ref119] Mann J. B.; Meek T. L.; Allen L. C. Configuration Energies of the Main Group Elements. J. Am. Chem. Soc. 2000, 122, 2780–2783. 10.1021/ja992866e.

[ref120] Mann J. B.; Meek T. L.; Knight E. T.; Capitani J. F.; Allen L. C. Configuration Energies of the d-Block Elements. J. Am. Chem. Soc. 2000, 122, 5132–5137. 10.1021/ja9928677.

