# Supplementary material for: Band Alignments, Electronic Structure, and Core-Level Spectra of Bulk Molybdenum Dichalcogenides (MoS2, MoSe2, and MoTe2)
Source: J Phys Chem C Nanomater Interfaces. 2022 Dec 1;126(49):21022–33. doi: 10.1021/acs.jpcc.2c05100 (PMC9761681; doi:10.1021/acs.jpcc.2c05100)
Supplement: Supplementary file 1 — jp2c05100_si_001.pdf [file jp2c05100_si_001.pdf]

Band Alignments, Electronic Structure, and Core Level Spectra, of Bulk Molybdenum Dichalcogenides (MoS_2, $MoSe_2$, and $MoTe_2$): Supplementary Information

Leanne A. H. Jones,[†] Zongda Xing,[‡] Jack E. N. Swallow,[†] Huw Shiel,[†] Thomas J. Featherstone,[†] Matthew J. Smiles,[†] Nicole Fleck,[†] Pardeep K. Thakur,[¶] Tien-Lin Lee,[¶] Laurence J. Hardwick,[§] David O. Scanlon,[‡] Anna Regoutz,[‡] Tim D. Veal,[*,†] and Vinod R. Dhanak[†]

†*Stephenson Institute for Renewable Energy and Department of Physics, University of Liverpool, Liverpool, L69 7ZF, UK*

‡*Department of Chemistry, University College London, 20 Gordon Street, London, WC1H 0AJ, UK*

¶*Diamond Light Source Ltd., Diamond House, Harwell Science and Innovation Campus, Didcot, Oxfordshire OX11 0DE, UK*

§*Stephenson Institute for Renewable Energy and Department of Chemistry, University of Liverpool, Liverpool, L69 7ZF, UK*

E-mail: T.Veal@liverpool.ac.uk

Table S1: Literature values of the experimental band gaps of MoS_2, $MoSe_2$ and $MoTe_2$ in comparison to the theoretical values from this work.

	Direct (This work, eV)	Exp. Direct (eV)	Indirect (This work, eV)	Exp. indirect (eV)
MoS_2	1.450	1.37[1]<br>1.17[3]	2.008	1.932[2]<br>1.87[1]
$MoSe_2$	1.338	1.25[1]<br>1.11[3]<br>1.165[5]	1.794	1.50[4]<br>1.56[1]
$MoTe_2$	1.057	0.89[1]	1.345	1.15[6]<br>1.06[1]

Table S2: The area ratios used for the corresponding core levels measured using 1.0 and 5.9 keV photons which were deduced by the ratio of the Scofield photoionisation cross-sections of each doublet.

Orbitals	Area ratio	
	1.0 keV	5.9 keV
Mo $3d_{3/2}$, Mo $3d_{5/2}$	0.68	0.71
S $2p_{1/2}$, S $2p_{3/2}$	0.51	0.51
Se $3d_{3/2}$, Se $3d_{5/2}$	0.68	0.70
Te $3d_{3/2}$, Te $3d_{5/2}$	0.68	0.72

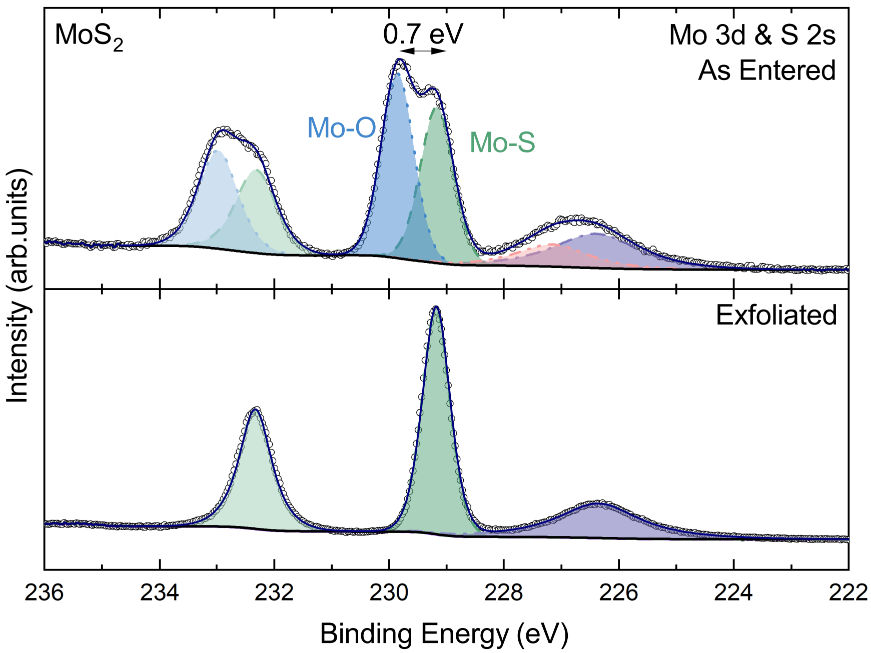

Figure S1: The XPS Mo 3d core level spectra of as-entered and exfoliated MoS_2. The higher binding energy component is attributed to an oxide and lies 0.7 eV above the peak for MoS_2.

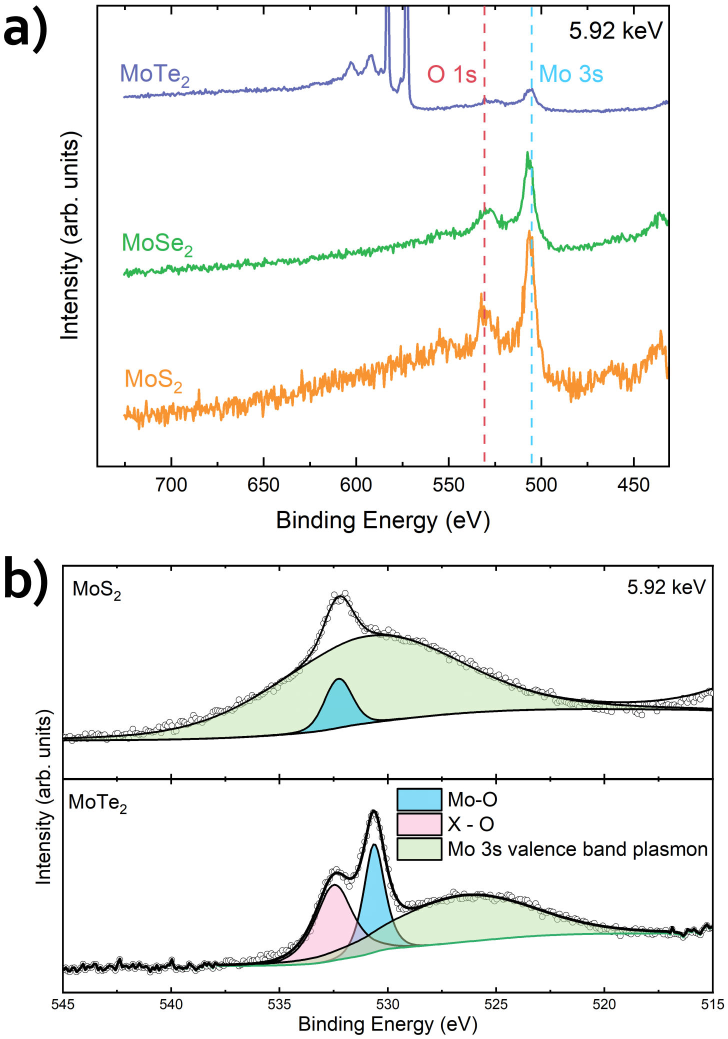

Figure S2: a) Survey spectra of the *in-situ* cleaved MoS_2, $MoSe_2$ and $MoTe_2$ depicting the weak signal of O 1*s*. b) *ex-situ* cleaved O 1*s* spectra for MoS_2 and $MoTe_2$.

Figure S2 presents the survey spectra of the *in-situ* cleaved crystals highlighting the effectiveness of the cleaving process. The O 1*s* core level overlaps heavily with the valence band plasmon of the Mo 3*s*, however, when present the O 1*s* is still visible. From Figure S2 a), the cleaving of $MoSe_2$ is seen to have completely remove any oxide contamination. For MoS_2 and $MoTe_2$ an extra intensity in the form of a sharp feature within the Mo 3*s* valence band plasmon can be seen which is due to a small amount of oxide present post *in situ* cleaving. For $MoTe_2$, there are two O peaks which are attributed to MoO_2 and TeO_2 whereas MoS_2 only has one resolvable peak which is indicative of Mo-O.

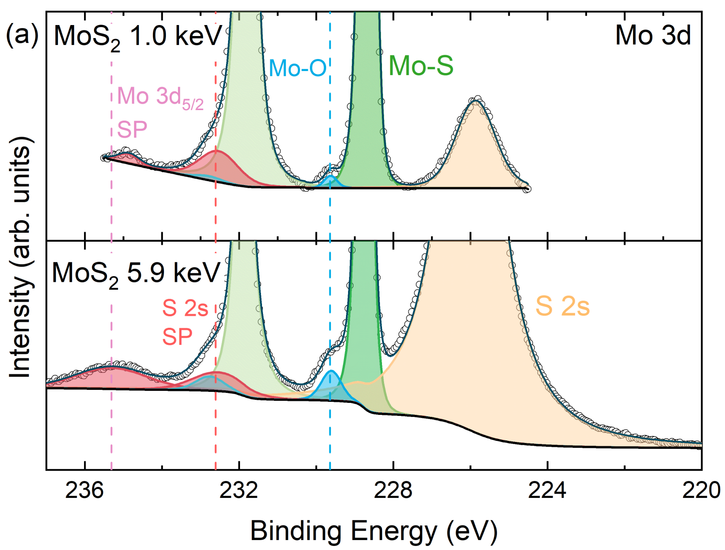

Figure S3: Zoomed in version of Figure 4 a) in the main text to elucidate the weak features.

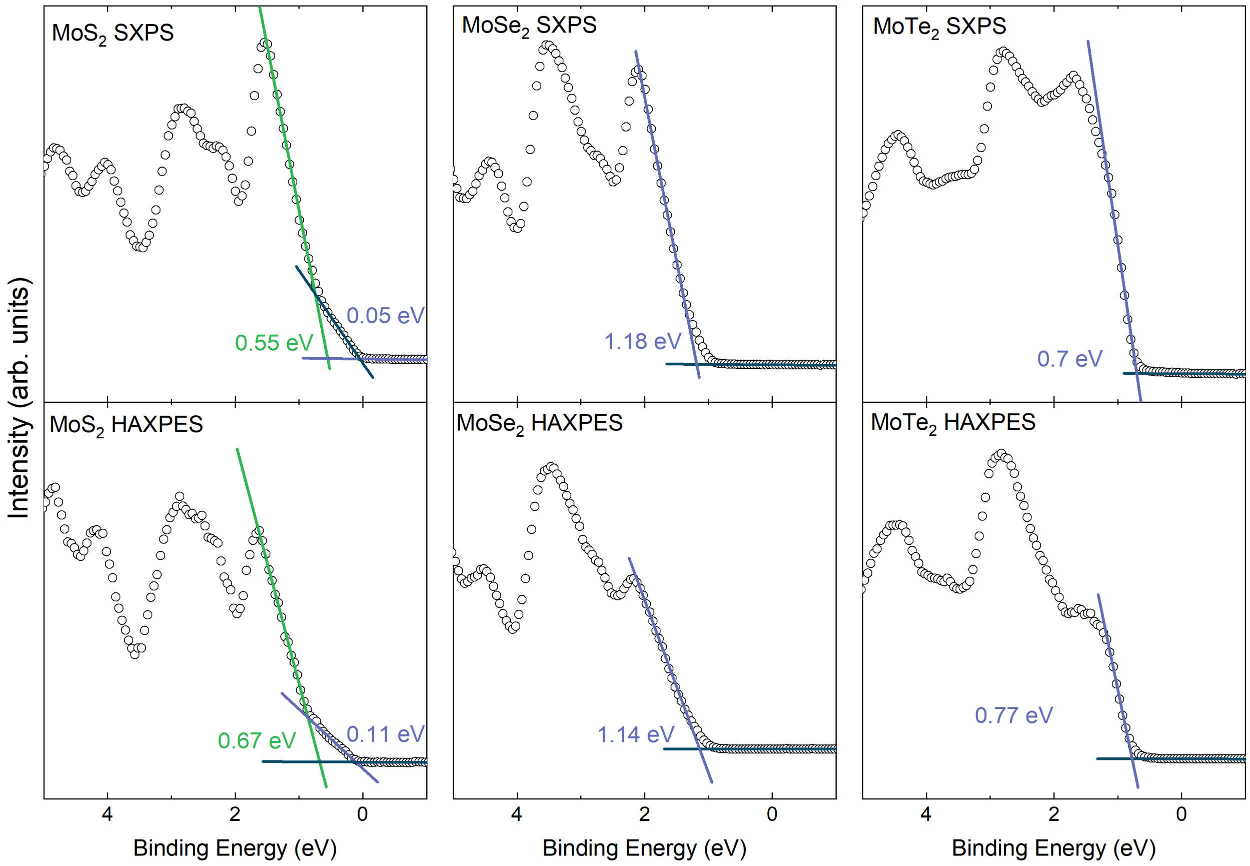

Figure S4: Valence band maximum determination using the extrapolation method for all three dichalcogenides of the MoX_2 series for both SXPS and HAXPES measurements. The MoS_2 SXPS and HAXPES spectra have extrapolations of two different “edges” which give different VBM positions as discussed in the main manuscript.

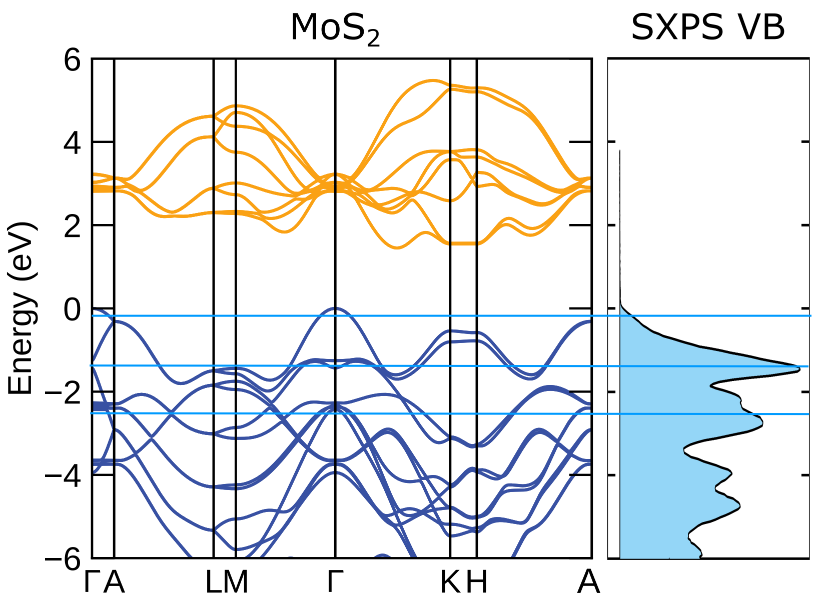

Figure S5: Comparison of the DFT-calculated band diagram of MoS_2 with the experimental valence band photoemission spectrum.

Figure S5 shows the SXPS valence band aligned to the calculated band diagram. At the VBM of the band diagram it can be seen that there is a region with a low density of states. This translates to a weak feature at the valence band edge in the experimental data. When looking at -1 eV in the band structure, a region of high density of states can be seen which aligns to the first peak of the valence band spectrum. Therefore, the weak feature in SXPS and HAXPES measurement was decided to be the VBM feature.

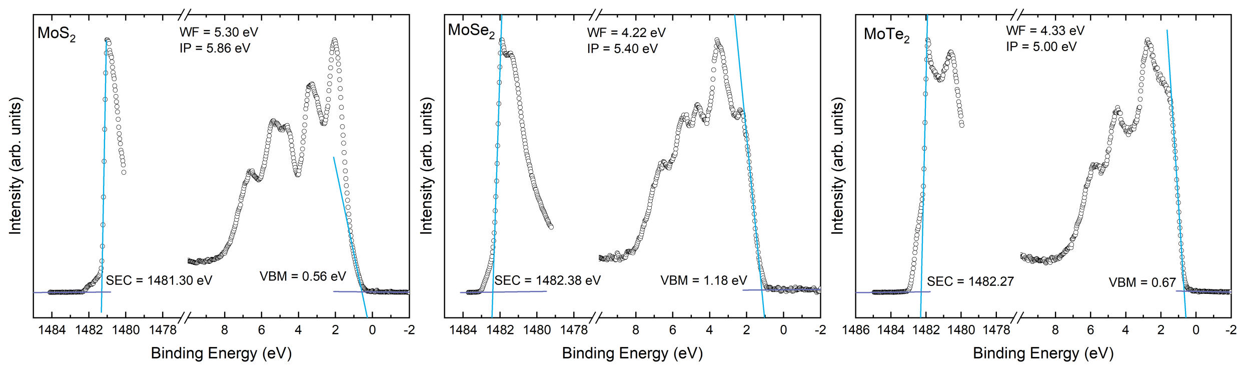

Figure S6: Valence band maximum and secondary electron cutoff measurements for all three dichalcogenides using lab-based XPS.

Figure S6 shows the valence band and secondary electron cutoff measurements of MoS_2, $MoSe_2$ and $MoTe_2$. As for the SXPS and HAXPES, the weaker feature at the valence band edge of MoS_2 was taken as the valence band maximum (VBM) for the XPS measurements. The VBM values agree between the lab-based XPS and the SXPS and HAXPES measurement for both $MoSe_2$ and $MoTe_2$. There is a difference in binding energy with respect to the Fermi level of ∼0.5 eV between the SXPS/HAXPES and the lab-based XPS VBM value. This could be caused by a difference in the surface preparation between the two measurements as the exfoliation technique used here left residual C on the surface from the carbon tape used. It has been reported that surface electron accumulation can be present at the surface of MoS_2, depending on the surface preparation.[7] However, to obtain the ionisation potential (IP) value, the position of the VBM with respect to the Fermi level is not important. If the Fermi level position changes, it does not alter the IP value as it is the VBM to vacuum level separation.

Table S3: Literature values of the work function (WF) and ionisation potentials (IP) of MoS_2, $MoSe_2$ and $MoTe_2$.

	Sample details	WF (eV)	IP (eV)	Reference
MoS_2	CVD grown on SiO_2/Si	N/A	5.77	[8]
	CVT grown single crystal exfoliated at 10^{-6} mbar	4.8	5.6	[9]
	CVD grown	4.45		[10]
	Single crystal	5.42	6.09	[11]
	Not specified	4.92		[12]
	Single crystal		5.47	[13]
$MoSe_2$	CVD grown on SiO_2/Si		5.34	[8]
	$MoSe_2$ grown on Bi_2Se_3	4.35	5.42	[14]
	CVT grown single crystal exfoliated at 10^{-6} mbar	4.4	5.5	[9]
$MoTe_2$	CVT grown single crystal exfoliated at 10^{-6} mbar	4.1	5.0	[9]
	CVT grown single crystal	4.35	4.95	[15]
	Single crystal	3.8		[16]
	Single crystal		5.13	[13]

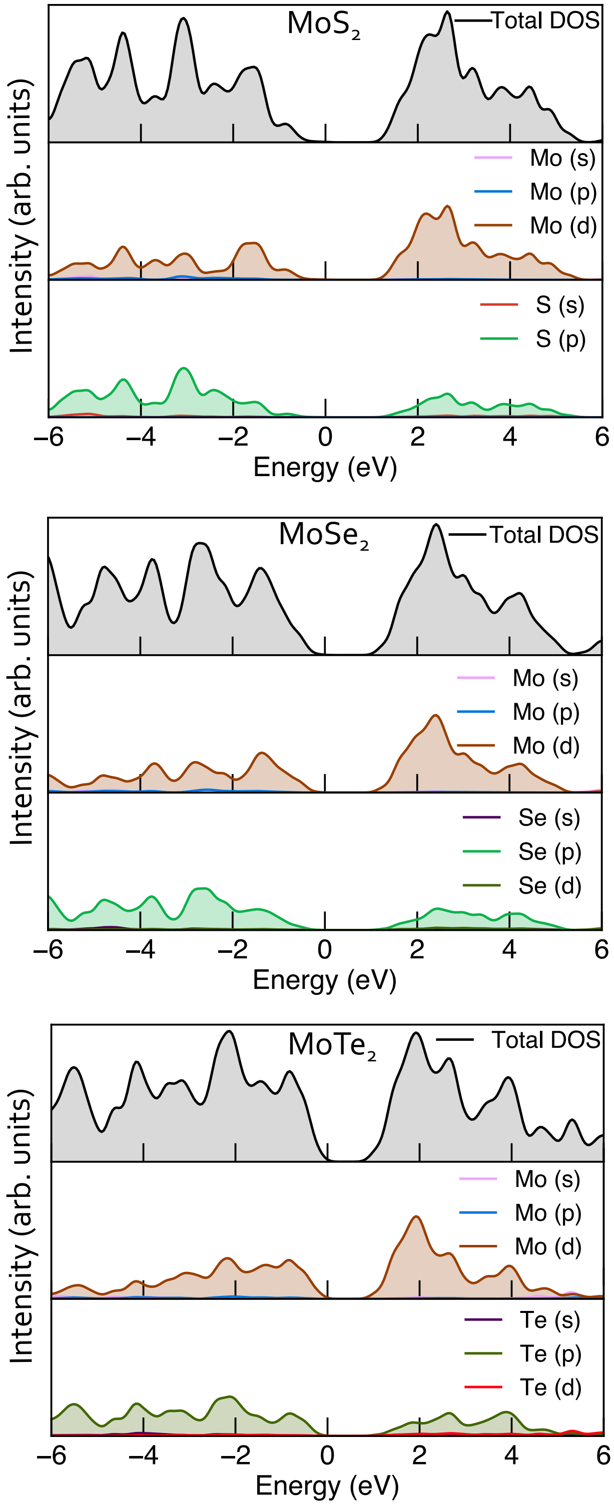

Figure S7: The uncorrected partial density of states.

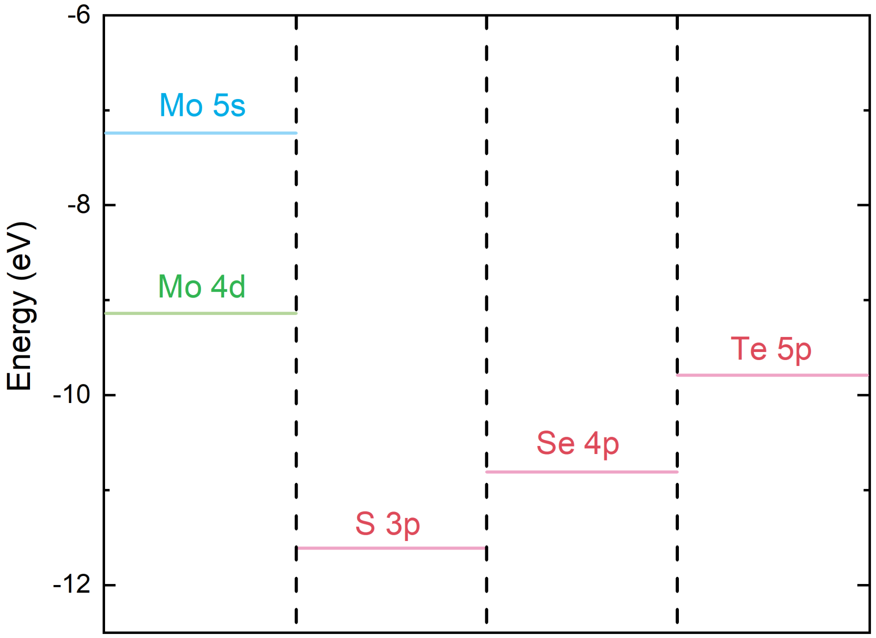

Figure S8: Configuration energies of the Mo 4d, S 3p, Se 4p and Te 5p orbitals from the calculations of Mann[17,18]

Figure S8 shows the configuration energies of the Mo 4d and chalcogen p levels. This shows that down the chalcogen series the energy of the p orbital becomes closer to that of the Mo 4d which in turn increases the mixing between the two orbitals.

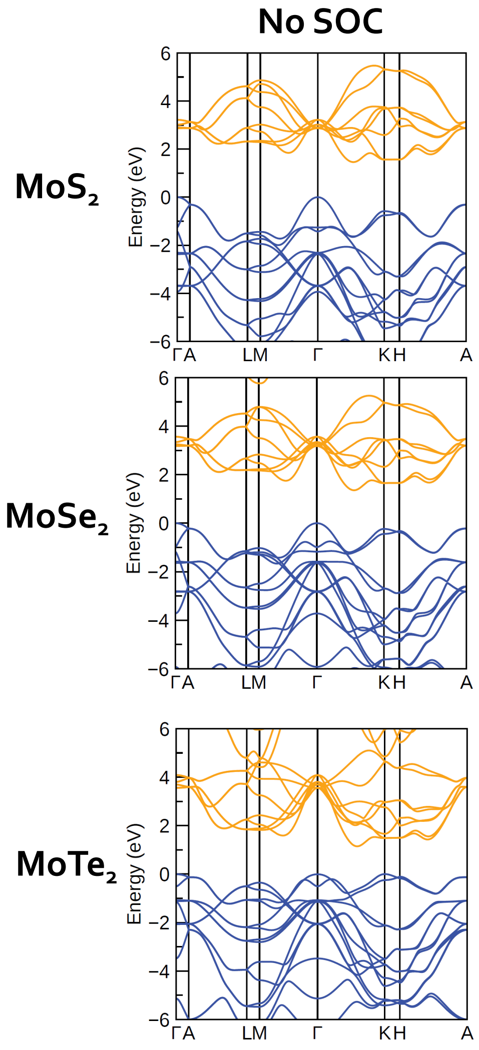

Figure S9: The calculated band structure showing the effect of including spin-orbital coupling.

In order to calculate the electronic properties on the highest accuracy level we chose to use the HSE06 hybrid functional. As for the inclusion of spin-orbital coupling (SOC), despite that S and Se being light element therefore the SOC effect is minor, Te being a period 5 elements will see a relatively larger impact. For the completeness of the study we decided to take the SOC into consideration. We have run the calculations without the SOC and from the results we can spot some differences, especially in $MoTe_2$ as presented in Figure S9. While the SOC did not change the overall band structure of MoX_2 in a meaningful way the inclusion of SOC has reduced the band gap in MoS_2, $MoSe_2$ and $MoTe_2$ by 0.008 eV, 0.018 eV and 0.088 eV respectively. With PBE+D3 the band gap difference is much larger as DFT methods such as PBE (either with or without D3 correction) tend to underestimate the band gap value by a large margin and therefore less relevant for this study. The band gap calculated by PBE+D3 is smaller by 0.561 eV, 0.515 eV, 0.412 eV for MoS_2, $MoSe_2$ and $MoTe_2$ respectively compared to the HSE06+SOC.

Table S4: Band gap using different functionals in eV.

	MoS_2	$MoSe_2$	$MoTe_2$
PBE+D3	0.889	0.872	0.733
HSE06	1.458	1.356	1.145
HSE06+SOC	1.45	1.338	1.057

References

(1) Zelewski, S. J.; Kudrawiec, R. Photoacoustic and modulated reflectance studies of indirect and direct band gap in van der Waals crystals. *Sci. Rep.* **2017**, *7*, 15365.

(2) Kopaczek, J.; Zelewski, S. J.; Polak, M. P.; Gawlik, A.; Chiappe, D.; Schulze, A.; Caymax, M.; Kudrawiec, R. Direct and indirect optical transitions in bulk and atomically thin MoS_2 studied by photoreflectance and photoacoustic spectroscopy. *J. Appl. Phys.* **2019**, *125*, 135701.

(3) Goldberg, A. M.; Beal, A. R.; Lévy, F. A.; Davis, E. A. The low-energy absorption edge in 2H-MoS_2 and 2H-$MoSe_2$. *Philos. Mag.* **1975**, *32*, 367–378.

(4) Choi, B. K.; Kim, M.; Jung, K.-H.; Kim, J.; Yu, K.-S.; Chang, Y. J. Temperature dependence of band gap in $MoSe_2$ grown by molecular beam epitaxy. *Nanoscale Res. Lett.* **2017**, *12*, 492.

(5) Hu, S.; Lee, Y.; Shen, J.; Chen, K.; Tiong, K.; Huang, Y. Temperature dependence of absorption edge anisotropy in 2H-$MoSe_2$ layered semiconductors. *Solid State Commun.* **2006**, *139*, 176–180.

(6) Lezama, I. G.; Arora, A.; Ubaldini, A.; Barreteau, C.; Giannini, E.; Potemski, M.; Morpurgo, A. F. Indirect-to-Direct Band Gap Crossover in Few-Layer $MoTe_2$. *Nano Lett.* **2015**, *15*, 2336–2342.

(7) Siao, M. D.; Shen, W. C.; Chen, R. S.; Chang, Z. W.; Shih, M. C.; Chiu, Y. P.; Cheng, C.-M. Two-dimensional electronic transport and surface electron accumulation in MoS_2. *Nat. Commun.* **2018**, *9*, 1442.

(8) Keyshar, K.; Berg, M.; Zhang, X.; Vajtai, R.; Gupta, G.; Chan, C. K.; Beechem, T. E.; Ajayan, P. M.; Mohite, A. D.; Ohta, T. Experimental Determination of the Ionization Energies of $MoSe_2$, WS_2, and MoS_2 on SiO_2 Using Photoemission Electron Microscopy. *ACS Nano* **2017**, *11*, 8223–8230.

(9) Shimada, T.; Ohuchi, F. S.; Parkinson, B. A. Work Function and Photothreshold of Layered Metal Dichalcogenides. *Japanese J. Appl. Phys.* **1994**, *33*, 2696–2698.

(10) Lee, S. Y.; Kim, U. J.; Chung, J.; Nam, H.; Jeong, H. Y.; Han, G. H.; Kim, H.; Oh, H. M.; Lee, H.; Kim, H.; *et al.*, Large Work Function Modulation of Monolayer MoS_2 by Ambient Gases. *ACS Nano* **2016**, *10*, 6100–6107.

(11) Diaz, H. C.; Ma, Y.; Chaghi, R.; Batzill, M. High density of (pseudo) periodic twin-grain boundaries in molecular beam epitaxy-grown van der Waals heterostructure: $MoTe_2/MoS_2$. *Appl. Phys. Lett.* **2016**, *108*, 191606.

(12) Lin, J.; Zhong, J.; Zhong, S.; Li, H.; Zhang, H.; Chen, W. Modulating electronic transport properties of MoS_2 field effect transistor by surface overlayers. *Appl. Phys. Lett.* **2013**, *103*, 063109.

(13) Schlaf, R.; Lang, O.; Pettenkofer, C.; Jaegermann, W. Band lineup of layered semiconductor heterointerfaces prepared by van der Waals epitaxy: Charge transfer correction term for the electron affinity rule. *J. Appl. Phys.* **1999**, *85*, 2732–2753.

(14) Yang, J.; Wang, C.; Ju, H.; Sun, Y.; Xing, S.; Zhu, J.; Yang, Q. Integrated Quasiplane Heteronanostructures of $MoSe_2/Bi_2Se_3$ Hexagonal Nanosheets: Synergetic Electrocatalytic Water Splitting and Enhanced Supercapacitor Performance. *Adv. Funct. Mater.* **2017**, *27*, 1703864.

(15) Williams, R. H. The structure of the upper valence bands in $MoTe_2$ and $NbSe_2$. *J. Phys. C: Solid State Phys.* **1973**, *6*, L32–L35.

(16) Schlaf, R.; Tiefenbacher, S.; Lang, O.; Pettenkofer, C.; Jaegermann, W. Van der Waals epitaxy of thin InSe films on $MoTe_2$. *Surf. Sci.* **1994**, *303*, L343 – L347.

(17) Mann, J. B.; Meek, T. L.; Allen, L. C. Configuration Energies of the Main Group Elements. *J. Am. Chem. Soc.* **2000**, *122*, 2780–2783.

(18) Mann, J. B.; Meek, T. L.; Knight, E. T.; Capitani, J. F.; Allen, L. C. Configuration Energies of the d-Block Elements. *J. Am. Chem. Soc.* **2000**, *122*, 5132–5137.
